# Ligand chain length drives activation of lipid G protein-coupled receptors

**DOI:** 10.1038/s41598-017-02104-5

**Published:** 2017-05-17

**Authors:** Anastassia Troupiotis-Tsaïlaki, Julian Zachmann, Inés González-Gil, Angel Gonzalez, Silvia Ortega-Gutiérrez, Maria L. López-Rodríguez, Leonardo Pardo, Cedric Govaerts

**Affiliations:** 1 Laboratoire de Structure et Fonction des Membranes Biologiques, Université Libre de Bruxelles, Brussels, Belgium; 2 Laboratori de Medicina Computacional, Unitat de Bioestadística, Facultat de Medicina, Universitat Autònoma de Barcelona, 08193 Bellaterra, Barcelona Spain; 3 Departamento de Química Orgánica I, Facultad de Ciencias Químicas, Universidad Complutense de Madrid, E-28040 Madrid, Spain

## Abstract

Sphingosine-1-phosphate (S1P) is a lipid mediator that can activate five cell membrane G protein-coupled receptors (GPCRs) which carry a variety of essential functions and are promising drug targets. S1P is composed of a polar zwitterionic head-group and a hydrophobic alkyl chain. This implies an activation mechanism of its cognate receptor that must be significantly different from what is known for prototypical GPCRs (ie receptor to small hydrophilic ligands). Here we aim to identify the structural features responsible for S1P agonism by combining molecular dynamics simulations and functional assays using S1P analogs of different alkyl chain lengths. We propose that high affinity binding involves polar interactions between the lipid head-group and receptor side chains while activation is due to hydrophobic interactions between the lipid tail and residues in a distinct binding site. We observe that ligand efficacy is directly related to alkyl chain length but also varies with receptor subtypes in correlation with the size of this binding pocket. Integrating experimental and computational data, we propose an activation mechanism for the S1P receptors involving agonist-induced conformational events that are conserved throughout class A GPCRs.

## Introduction

**Figure 1:**
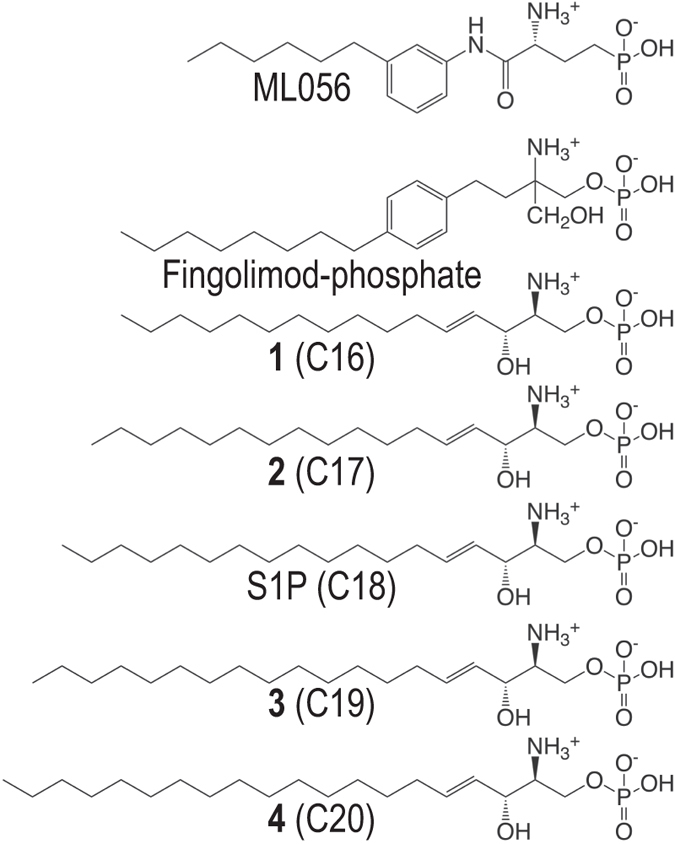
Structure of ML056, Fingolimod-phosphate, S1P and synthetic derivatives 1–4 of variable chain length.

By ensuring the conversion of extracellular stimuli into cellular responses, G protein-coupled receptors (GPCRs) modulate signaling pathways in a wide variety of biological processes. The diversity of GPCRs functions is associated with a remarkable variety in their cognate ligands, from both structural and chemical standpoints. Indeed, these receptors bind entities as different as calcium ions, small organic molecules (amines, steroids), nucleotides, peptides, proteins or lipids^1^. GPCRs are classified into five main families or classes based on sequence similarity^2^, the class A, also known as rhodopsin family, being the largest and most studied. High-resolution structures of receptors from different families have confirmed that GPCRs share a similar architecture of seven transmembrane (TM) α-helices forming a bundle and that the TM domains are structurally conserved^3, 4^. Class A GPCRs exhibit a distinctive feature that most of their ligands bind to a cavity inside the TM helices. While most of them recognize small polar agonists, GPCRs for lipid mediators are activated by hormone-like signaling molecules derived from lipid species, which possess long hydrophobic moieties^5^. This subfamily is mostly composed of the sphingosine-1-phosphate (S1P) and lysophosphatidic acid (LPA) receptors (formerly grouped in the endothelial differentiation gene or EDG family) as well as the cannabinoid receptors. Sphingosine-1-phosphate (S1P) regulates a plethora of biological functions in the central nervous system, immune and cardiovascular systems as well as numerous pathophysiological processes^6^. This lysophospholipid is produced intracellularly from sphingolipid catabolism and exerts its functions mainly via activation of five cell membrane specific GPCRs, S1P_1_-_5_ (initially edg-1, 3, 5, 6, and 8)^7^. The diversity of S1P signaling and its regulation stems from synthesis and degradation balance, differential expression of S1P receptors in various cell types as well as from their distinct coupling to G proteins subtypes^8^. S1P receptors display a variety of essential functions while activated by a unique agonist, conferring them a major therapeutic potential, as underlined by the approval by the US FDA of the S1P_1_-mediated immune modulator FTY720 (fingolimod; Gilenya^TM^, Novartis) for the treatment of relapsing-remitting multiple sclerosis^9, 10^. Fingolimod is a prodrug that is phosphorylated to form fingolimod-phosphate (Fig. 1), which activates lymphocyte S1P_1_ and prevents lymphocyte egress from lymphoid tissues^11^.

S1P is an amphipathic molecule composed of a polar zwitteronic head-group and a hydrophobic alkyl chain (Fig. 1). Several studies combining molecular modeling and mutagenesis investigating the binding of S1P to S1P receptors show that the agonist head-group interacts with charged residues at one site of the receptor and its alkyl chain lies at a hydrophobic binding pocket^6^. The antagonist-bound S1P_1_ receptor structure^12^ and molecular dynamics (MD) simulations of the process of ligand entry from the membrane bilayer to the binding pocket^13^ have confirmed the role of the ligand head-group in both the process of ligand entry and receptor recognition. The S1P_1_ antagonist ML056 polar moiety possesses a primary amine and phosphonate group, features that are closely related to those of S1P (Fig. 1). Though the role of ligand polar moiety has been investigated for the native agonist^14^ as well as for synthetic molecules^15^, the implication of the alkyl chain in receptors activation remains elusive. While the S1P head-group is required for its high affinity binding to the receptor, acting as an anchor, elements suggest that it is not key in receptor activation, but rather that the alkyl chain may be involved in agonist activity^16^. This assumption is supported by the fact that an antagonist head-group can share closely related chemical functions with S1P and interact via the same residues, as exemplified by ML056^12^. The hypothesis that agonism could depend on the deformation of the TM pocket induced by a hydrophobic volume is underpinned by the investigation of S1P_1_ interactions with a series of synthetic agonists^17^, as well as structure-activity relationships work on FTY720 analogs^18, 19^. The latter studies reveal that the change of alkyl substituent position from para- to meta- on the aromatic ring converts the agonist to an antagonist, whereas changing chain length from 6 to 10 carbons on the meta- analog restores agonist activity.

In this work, by combining computational and experimental approaches, we identify the structural and chemical features that are responsible for S1P agonism, leading to a comprehensive activation mechanism for the S1P receptor family.

## Results and Discussion

### Molecular dynamics simulations of S1P_1_ in complex with the natural S1P agonist

We first aimed at identifying the molecular interactions between the natural agonist S1P and its cognate receptor, using the crystal structure of the S1P_1_ receptor in complex with the antagonist ML056^12^ (Supplementary Fig. S1A), which bears significant chemical and structural similarities with S1P (Fig. 1). We performed unbiased 2 μs MD simulation of S1P_1_, in the inactive conformation as determined in the crystal structure, with the natural S1P agonist bound to the receptor (see Experimental Procedures). The zwitterionic head-group of S1P was docked into the receptor analogously to the binding mode of the co-crystallized ML056 antagonist, interacting with N101^2.60^, R120^3.28^ and E121^3.29^ as previously determined by site-directed mutagenesis^14^. The alkyl chain inserts into a hydrophobic/aromatic pocket between TMs 3, 5 and 6 (Supplementary Fig. S1B), similarly to previous proposed models^12, 20^. This docking pose suggests that the end-terminal tail of the ligand points either toward TMs 3 and 5 (mainly F125^3.33^, L128^3.36^, C206^5.43^, F210^5.47^ shown in light green spheres) or toward TMs 5 and 6 (T207^5.44^, F210^5.47^, F273^6.52^, L276^6.55^ shown in dark green spheres). However, as shown in Supplementary Fig. S1B, these side chains are highly packed together in the crystal structure of inactive S1P_1_. This suggests that the additional methylene units of the S1P requires modification of the conformation of these side chains and the packing of TMs 3, 5, or/and 6 for agonist binding and thus receptor activation (see below). This assumption seems likely since the side chains at positions 3.36^21, 22^, 5.43^23^, 5.47^24, 25^, 6.52^26^ and 6.55^27^ have been proposed to be involved in receptor activation in other members of the class A GPCR family. We have monitored the influence of S1P on dihedral χ1 angles of these side chains (red dots) during the MD simulation (Supplementary Fig. S1E). Importantly, no significant changes in the conformation of the side chains are observed. Thus, these results suggest that the additional length of the alkyl chain of the S1P agonist, relative to the ML056 antagonist, has not been capable to modify the packing of TMs 3, 5, and 6 in the MD simulation of inactive S1P_1_. However a plot of the root-mean-square-deviation (rmsd) of S1P’s zwitterionic head-group relative to the initial, ML056-like binding pose (Supplementary Fig. S1E, red line) clearly shows the head-group shifts out from the initial binding position as revealed by rmsd values >5 Å (see Supplementary Fig. S1C). In contrast, rmsd values of the TM domain of the receptor (broken red line in Supplementary Fig. S1E) remain low, indicating no significant conformational changes of the S1P_1_ structure. These results suggest that S1P cannot be accommodated by an inactive structure of S1P_1_ while maintaining the key interactions with N101^2.60^, R120^3.28^ and E121^3.29^.

**Figure 2:**
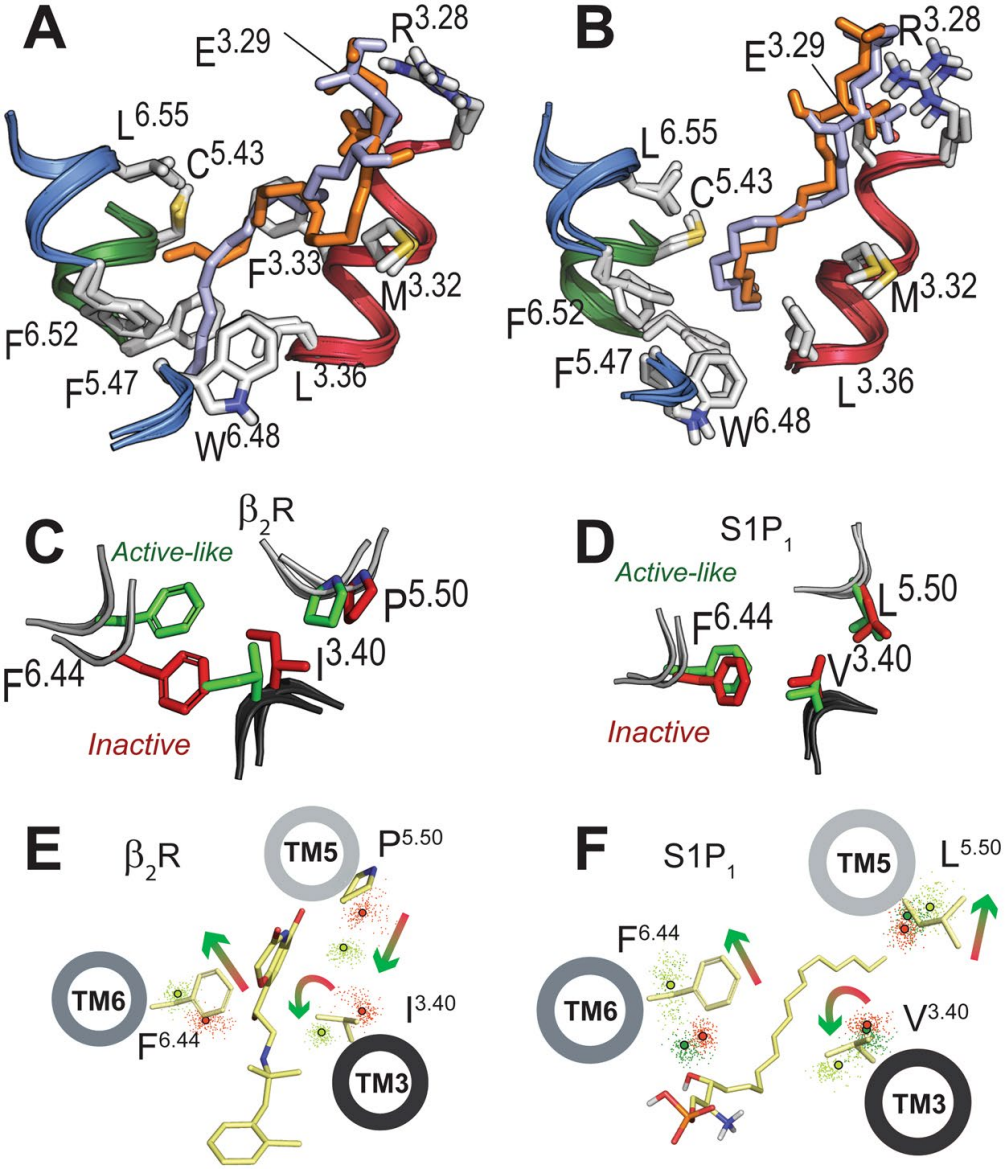
Mechanism of S1P-induced activation of S1P_1_. (**A**) Detailed view of the extracellular domain of the energy-minimized inactive-like and active-like models of S1P_1_. The initial binding pose of S1P used both in the inactive- and active-like MD simulation is shown in orange. A different conformation of the alkyl chain of S1P (in light blue) was also simulated for the active-like model of S1P_1_. (**B**) The final conformation, at 0.5 μs, obtained in the MD simulations of both starting conformations of S1P in complex with the active-like model of S1P_1_. (**C**,**D**) The starting positions of I121^3.40^, P211^5.50^ and F282^6.44^ (**C**) and V132^3.40^, L213^5.50^ and F265^6.44^ (**D**) (‘transmission switch’) used in the MD simulations of inactive- (in red) and active- (in green) conformations of the β_2_-adrenergic receptor (**C**) and S1P_1_ (**D**). Because the extracellular part of the active-like model of S1P_1_ is comparable to the inactive model (see Fig. 2A and Supplementary Fig. S2D), initial positions of these side chains, in the simulations, are similar and correspond to the inactive conformation. (**E**,**F**) Evolution of the Cβ atoms (dots) of I121^3.40^, P211^5.50^ and F282^6.44^ of β_2_- (**E**) and V132^3.40^, L213^5.50^ and F265^6.44^ of S1P_1_ (**F**) during the MD simulations (0.5 μs) of inactive- (in red) and active- (in light and dark green) like conformation of β_2_- in complex with the BI167107 agonist (light green, panel E) and S1P_1_ in complex with S1P (light green, panel F) and ligand-free (dark green, panel F). Centroids (calculated from 250 snapshots) of the Cβ atoms of these side chains are shown in red (inactive), dark green (ligand-free active conformation) and light green (agonist-bound active conformation) circles. Displayed helices, side chains, and agonists are shown for clarity. Arrows represent the observed movement of the helices during the MD simulations.

GPCRs are dynamic proteins that permit rapid small-scale structural fluctuations and pass through an energy landscape to adopt a number of conformations ranging from inactive to active^28^. However, pioneering work by the Kobilka group has shown that an agonist alone is not capable to stabilize the fully active conformation of the receptor in the absence of the G protein^29^. Similarly, MD simulations of an agonist bound to the inactive state of the receptor are not capable to reach active-like conformations in the absence of the G protein. Thus, to study S1P_1_ activation by S1P, we built an active-like model of S1P_1_ using the crystal structure of a nanobody-stabilized active state of the β_2_-adrenergic receptor^23^ as a template (see Experimental Procedures). In this model the intracellular part of S1P_1_ has been constructed as in the active conformation of β_2_-receptor (see Supplementary Fig. S2), in which the intracellular cavity for G protein binding is opened through the movement of the cytoplasmic end of TM 6 away from TM 3 and towards TM 5. Analogously to the structure of β_2_-receptor, the active-like model of S1P_1_ includes the G-protein-mimetic nanobody^30, 31^. It is important to remark that the extracellular part, including receptor side chain conformations, of the active-like model of S1P_1_ is similar to the inactive model (Supplementary Fig. S2D). For the active-like model, two independent replicas of 500 ns each were simulated, starting at different docking poses of the alkyl chain of S1P (Fig. 2A). Analysis of the trajectories revealed that these predicted complexes were highly stable, both the S1P ligand and the nanobody bound to the receptor (Supplementary Fig. S3). Thus, in contrast to the simulation of inactive S1P_1_, ligand rmsd of the zwitterionic head-group of S1P remained low (Supplementary Fig. S1E), indicating that this part of the ligand remained bound to N101^2.60^, R120^3.28^ and E121^3.29^. More important, in the two simulations, the terminal part of the alkyl chain of the ligand orients toward a small cavity that is opened between TMs 3 and 5 (Fig. 2B). Figure 2B shows that, independently of the starting position, the final conformational of the alkyl chain of S1P is similar in the two MD simulations.

### Mechanism of S1P-induced activation of S1P_1_

We assume, as a working hypothesis, that activation of S1P_1_ involves rearrangement of the conserved ‘transmission switch’ made of the amino acids at positions 3.40, 5.50, and 6.44^32^. This conserved ‘transmission switch’ has been proposed for other GPCRs based on the fact that, for instance, a hydrogen bond interaction between agonists and TM 5 in β_1_-^33^ and β_2_-^23^ or the conformational change of inactive 11-cis retinal to the active 11-trans retinal in rhodopsin^34^ or agonist-binding to A_2_A^35^, stabilizes a receptor conformation that includes an inward movement of TM 5 at the highly conserved P^5.50^, relative to inactive structures. For comparison purposes with S1P_1_, we simulated this ‘transmission switch’ in the β_2_-adrenergic receptor, a prototypical GPCR, for which experimental structures of the inactive and active conformation of the receptor are available (Fig. 2C). Clearly, the inward movement of TM 5 is sterically competing with a bulky hydrophobic side chain at position 3.40, triggering a small counterclockwise rotation, viewed from the extracellular part, of TM 3^36^ (Fig. 2E). Finally, the rotation of TM 3 repositions the side chain of F^6.44^, facilitating the outward movement of TM 6 for receptor activation^23^ (Fig. 2E). Next we analyzed in the MD simulations of inactive- and active-like S1P_1_ receptor this ‘transmission switch’ by monitoring the rotation and displacement of TMs 3, 5 and 6 (Fig. 2F). It is important to note that, in contrast to the β_2_-adrenergic receptor (Fig. 2C), the initial position of the TMs and the key V132^3.40^, L213^5.50^ and F265^6.44^ side chains of S1P_1_ were similar in inactive- and active-like conformations (Fig. 2D). However, the MD simulations reveal different final position of the helices (Fig. 2F). Relative to the inactive simulation, in the active simulation we observe an extracellular outward movement of TM 5, while TM 3 performs the proposed counterclockwise rotation of 10° (Fig. 2F and Supplementary Fig. S1D) positioning V132^3.40^ towards TM 6, which leads to a steric exclusion of V132^3.40^ with the side chain of F265^6.44^ and the outward movement of TM 6 for receptor activation. Clearly, the extracellular outward movement of TM 5, away from TM 3, during S1P_1_ activation is different to the inward movement observed in the process of activation of “typical” GPCRs such as the β_2_-receptor (compare Fig. 2E,F). Conformational divergence is expected in TM 5 as GPCRs activated by lipid mediators (S1P, LPA and cannabinoid receptors) lack the highly conserved P^5.50^ present in other GPCRs, thus suggesting a local conformational specificity. Importantly, the V132^3.40^L, L213^5.50^G or F265^6.44^G mutations, performed by others^14^, decreased the S1P-induced Emax by 55%, 46%, or 28%, respectively, relative to wt. Thus, we can conclude that activation of S1P_1_ involves several structural elements of the ‘transmission switch’ present in the other members of the GPCR family.

It is important to discriminate the effect of the ligand at the extracellular domain and of the nanobody at the intracellular domain in triggering these conformational changes of receptor activation. Thus, we performed an additional 0.5 μs of MD simulations of the active-like model of S1P_1_ bound to the nanobody but in the absence of the ligand for comparison purposes (see Experimental Procedures). Clearly, the ‘transmission switch’ elements of receptor activation do not occur in the absence of the ligand (Fig. 2F, light green vs. dark green). Thus, our simulations indicate that the S1P ligand or the nanobody alone cannot activate S1P_1_ and the ‘transmission switch’ elements of receptor activation are only observed in the presence of both the ligand and the G protein-mimetic.

What are the initial structural changes of S1P-induced receptor activation responsible for the rearrangement of the ‘transmission switch’? Analysis of the MD simulations shows that the terminal part of the alkyl chain of the ligand (the additional methylene units present in the agonist) inserts into a small cavity between TMs 3 and 5 (Fig. 2B). This cavity is created following conformational changes of L128^3.36^ from *trans* to *gauche-* and F210^5.47^ from *gauche-* to *trans* (Supplementary Fig. S1E). We propose that the conformational changes of the L128^3.36^ and F210^5.47^ side chains, opening the cavity for the ligand tail, initiate activation of the ‘transmission switch’ via rigid body motions of TMs 3 and 5. F210^5.47^ is pointing toward TM 3 in the inactive *gauche-* conformation and toward TM 6 in the active *trans* conformation to interact with W269^6.48^, whereas L128^3.36^ is pointing toward TM 6 in the inactive *trans* conformation and toward TM 7 in the active *gauche-* conformation (Fig. 2A,B). Fujiwara *et al*. showed that the L128^3.36^G, F210^5.47^G or W269^6.48^A mutations impede S1P-induced receptor activation without modifying ligand binding^14^. Moreover, the role of these side chains at position 3.36^21, 22^, 5.47^24, 25^ or 6.48^21, 22, 24^ in receptor activation has previously been described for other GPCRs. Importantly, the MD simulation of S1P_1_ bound to the nanobody but in the absence of the ligand does not trigger the conformational changes of L128^3.36^ from *trans* to *gauche-* and F210^5.47^ from *gauche-* to *trans* (dark green data points distribute like red data points in Supplementary Fig. S1E). These findings reinforce our proposal that both the ligand and the intracellular nanobody are required for receptor activation.

Therefore our simulations suggest that the ability of the ligand to stabilize the active state of the S1P_1_ receptor is coupled to the insertion of the lipid tail inside the hydrophobic pocket between TMs 3, 5 and 6. We have subsequently tested this model by measuring activation of the receptor using S1P analogs of different chain length.

### Synthesis of S1P analogs

We synthesized S1P analogs **1**–**4** with decreasing and increasing number of methylene units (n = 10, 11, 13, 14) in the hydrophobic chain with respect to S1P (n = 12), while the head-group of the molecule was kept identical. The compounds were prepared as depicted in Fig. S4. Thus, the corresponding terminal alkenes were coupled to phosphorylated fragment **5** via an olefin cross metathesis reaction to obtain intermediates **6**–**9**. Then, treatment of these intermediates with bromotrimethylsilane afforded the desired sphingosine derivatives **1**–**4** by simultaneous deprotection of the amino and phosphate groups. S1P, with a total number of 18 carbon atoms (C18), and the synthesized derivatives of 16 (**1** or C16), 17 (**2** or C17), 19 (**3** or C19) and 20 (**4** or C20) carbon atoms are represented in Fig. 1.

### Functional characterization of S1P analogs at S1P receptors

**Figure 3:**
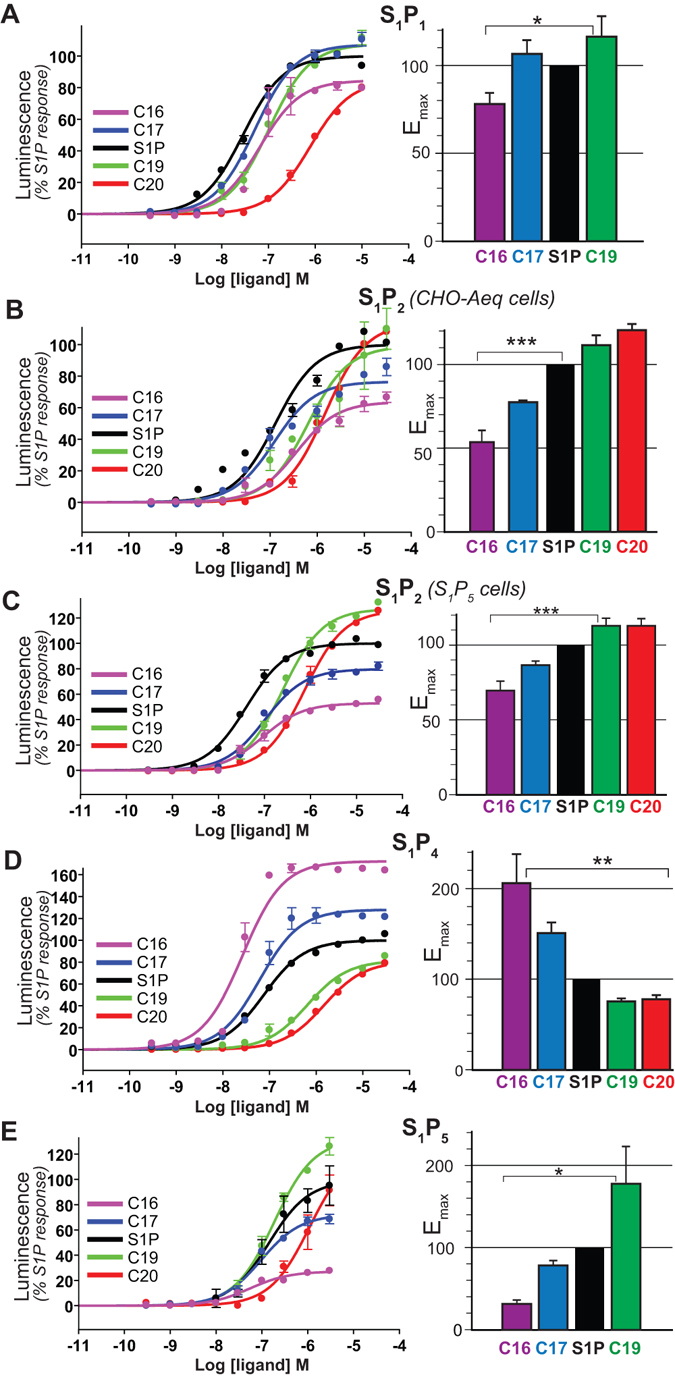
Functional response of S1P receptors to S1P synthetic analogs. Aequoscreen CHO-K1 cells expressing the different S1P receptors were subjected to stimulation with increasing concentrations of S1P-derived ligands and the resulting luminescence was measured. (**A**) S1P_1_, n = 4 (**B**) S1P_2_ from CHO-Aeq cells, n = 3 (**C**) S1P_2_ from S1P_5_ cells, n = 4 (**D**) S1P_4_, n = 4 (**E**) S1P_5_, n = 2. The corresponding dose-response curves for one representative experiment are shown, where each curve represents the mean ± S.E.M of duplicate data points. Luminescence intensities are normalized on maximal response to the natural agonist S1P. Histograms on the right show maximal efficacy (E_max_) values calculated for each S1P analog at S1P receptors, based on the sigmoidal fitting of aequorin functional response. The data plotted represent the mean ± S.E.M and are expressed in % of maximal efficacy in response to stimulation by S1P. Statistical significance was assessed using one-way ANOVA with a Scheffe’s *post hoc* test: *p < 0.05, **p < 0.01, ***p < 0.001.

In order to assess the influence of ligands chain length on the activation of S1P receptors, we compared the functional response induced by the synthesized S1P derivatives on mammalian cell lines expressing the receptor of interest (see Experimental Procedures). Figure 3A shows the dose-dependent curves corresponding to S1P_1_ functional responses. We observe that all the S1P analogs tested are agonists of the S1P_1_ receptor, but to different extents, both in terms of potency and efficacy. Mean S1P-normalized E_max_ (maximal efficacy) and pEC_50_ values of the different compounds towards S1P_1_ obtained from dose-response curves are presented in Tables 1 and 2, respectively. Clearly, the rank order of receptor activation (E_max_) is C19 (116% of S1P maximal activation) ≈ C17 ≈ C18 (S1P) > C16 (78%). For C20, due to the lower potency, E_max_ determination was no reliable within the range of concentrations tested and is thus not taken into account in the activation efficacy interpretation. The mean E_max_ values on 4 independent experiments for each compound are represented on the corresponding histogram of Fig. 3A, illustrating the reproducibility of the observed trend in maximal efficacy. The fact that at saturating concentration the different S1P analogs exhibit different maximal response clearly indicates that the alkyl chain is involved in stabilizing the active conformation of the receptor. Supplementary Fig. S6 shows the position of V132^3.40^, L213^5.50^ and F265^6.44^ (‘transmission switch’) obtained during MD simulations of S1P_1_ bound to these ligands. The rank order of E_max_ correlates with the movements of the ‘transmission switch’ amino acids. Clearly, the partial agonist C16 triggers the least outward movement of TM 5, rotation of TM 3, and the outward movement of TM 6 relative to the other full agonist ligands.

**Table 1:** E_max_ values of S1P derivatives at S1P receptors.

	C16	C17	S1P	C19	C20
S1P_1_ (n = 4)	78 ± 6	107 ± 8	100	117 ± 12	*n.r*.
S1P_2_^1^ (n = 4)	70 ± 6	87 ± 3	100	113 ± 1	113 ± 5
S1P_2_^2^ (n = 3)	54 ± 7	78 ± 1	100	112 ± 6	121 ± 4
S1P_4_ (n = 4)	206 ± 32	151 ± 12	100	76 ± 3	79 ± 4
S1P_5_ (n = 2)	32 ± 5	79 ± 6	100	178 ± 45	*n.r*.

The maximal efficacy for each compound at each S1P subtype was determined from the dose-response curves of the aequorin-based functional assay. Mean values on 2 to 4 independent experiments are normalized on S1P maximal response and expressed in %, along with the corresponding S.E.M. values.

^1^S1P_5_ cells, ^2^CHO-Aeq cells, *n.r*. = not reliable.

**Table 2:** pEC50 values of S1P derivatives at S1P receptors.

	C16	C17	S1P	C19	C20
S1P_1_ (n = 4)	6.5 ± 0.3	6.9 ± 0.2	7.1 ± 0.3	6.6 ± 0.2	*n.r*.
S1P_2_^1^ (n = 4)	6.9 ± 0.1	7.0 ± 0.1	7.5 ± 0.0	6.7 ± 0.1	6.3 ± 0.1
S1P_2_^2^ (n = 3)	6.5 ± 0.2	7.1 ± 0.1	7.3 ± 0.2	6.5 ± 0.2	6.0 ± 0.2
S1P_4_ (n = 4)	7.3 ± 0.2	7.1 ± 0.1	7.0 ± 0.1	6.1 ± 0.1	5.6 ± 0.1
S1P_5_ (n = 2)	7.4 ± 0.2	6.9 ± 0.1	7.3 ± 0.5	6.7 ± 0.1	*n.r*.

The experimental EC_50_ for all S1P derivatives were determined from the dose-response curves of the aequorin-based functional assays. Mean values of pEC_50_ (negative logarithm of EC_50_) on 2 to 4 independent experiments are presented along with the corresponding S.E.M. values.

^1^S1P_5_ cells, ^2^CHO-Aeq cells, *n.r*. = not reliable.

In order to further test this hypothesis, we extended our investigation to other receptors of the S1P family, by performing functional assays with the synthetic ligands on S1P_2_, S1P_4_, or S1P_5_ receptors. Figure 3 shows the dose-response curves obtained for the other sphingosine-1-phosphate receptors (S1P_2_, S1P_4_ and S1P_5_) upon stimulation by S1P and its synthetic derivatives. All compounds display positive efficacies for all the S1P receptor subtypes tested and thus behave like agonists at these receptors. The corresponding E_max_ and pEC_50_ values are shown in Tables 1 and 2. For S1P_2_ coming from CHO-Aeq cells (Fig. 3B), C20 is the more efficacious with 120% of S1P maximal response, followed by C19 at 112%, whereas C17 and C16 display a lower efficacy than S1P with respectively 78% and 54%. The reproducible trend of E_max_ values shows that the longer the alkyl chain, the higher the efficacy of the compound. For S1P_2_ in the CHO-Aeq system, we observed a non-specific contribution at high concentrations (unlikely due to another S1P receptor) that lowers the quality of fitting. Therefore we confirmed our S1P_2_ data using S1P_5_ cell line, knowing that about 90% of the functional response detected in these cells is inhibited by JTE013, a specific S1P_2_ antagonist, and thus is due to S1P_2_ receptors. In this system (Fig. 3C), C19 displays a higher E_max_ value than S1P of about 113%, whereas C17 displays lower E_max_ than S1P around 87%. Finally, C16 has the lowest efficacy with 70% of maximal S1P activation. Functional responses obtained on S1P_5_ cell line are exempt of non-specific contribution at high concentrations of ligands, within the range tested. As the two cell lines are transfected with distinct promiscuous G proteins, we can expect that the apparent potencies and efficacies can vary slightly from one system to another, even when looking at identical receptors. Nevertheless, we observe the same behavior in both systems: compounds with longer hydrophobic chain activate S1P_2_ receptor more efficiently (Fig. 3C). This agrees with the overall behavior observed with S1P_1_.

S1P_4_ activation profiles (Fig. 3D) display obvious differences with the previous S1P receptor subtypes tested. Strikingly, efficacy at the S1P_4_ receptor is 2 times higher for C16 than for S1P and 1.5 time for C17, while compounds C19 and C20 display E_max_ values between 75 and 80% of S1P maximal activation. Therefore, the activation trend observed is the opposite from the other subtypes with respect to S1P showing that shorter chains clearly favor S1P_4_ activation.

The S1P_5_ functional response was determined in the presence of the S1P_2_ specific antagonist JTE013, which allows to entirely remove the contribution of endogenous S1P_2_ receptor (Fig. 3E). The efficacies show that the longer chains activate better the receptor than the shorter ones, with C19 E_max_ estimation (~180%) higher than S1P, whereas C17 show 78% and C16 only 32% efficacy. Note that C19 E_max_ value could be underestimated given that the plateau was not clearly reached. As in the case of S1P_1_, the C20 response curve did not allow the determination of a reliable E_max_, due to its low EC_50_.

Tables 1 and 2 summarize the S1P-normalized E_max_ and pEC_50_ values obtained for S1P and all the synthetic derivatives at the four S1P receptor subtypes tested. As explained above, E_max_ determination for C20 on S1P_1_ and S1P_5_ receptors was not considered as reliable. By comparing efficacies, we can conclude that S1P_2_ and S1P_5_ are activated better by longer hydrophobic chains. S1P_1_ follows a similar trend, but with lower distinction between the compounds triggering the maximal activity C19, S1P and C17, while significantly lower efficacy is observed for C16. Interestingly, S1P_4_ presents the opposite trend, meaning that the shorter chains activate the system to the higher level than the longer ones.

Taken together, our results on G protein-triggered calcium mobilization support that the alkyl chain length is a key factor of agonism, an observation that can be applied to almost the whole S1P receptors family. In addition, functional properties are subtype-dependent within S1P receptors family, suggesting a specific behavior in response to hydrophobic chain length modulation.

**Figure 4:**
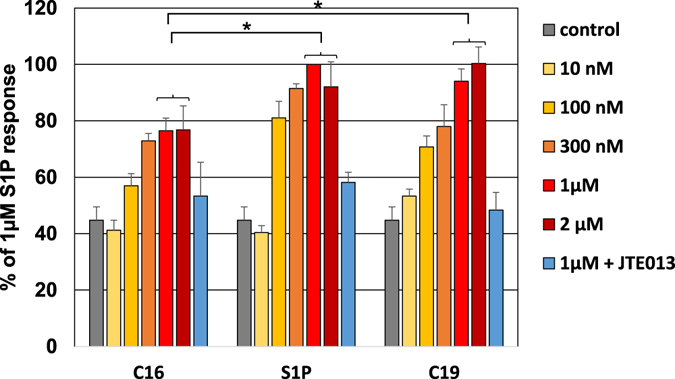
NF-κB activation *via* S1P_2_ in response to S1P analogs. Human BEAS-2B cells naturally expressing S1P_2_ receptor were subjected to stimulation with increasing concentrations (10 nM to 2 µM, from light yellow to dark red) of C16, S1P and C19 and resulting NF-κB activation was followed by luminescence (luciferase reporter). Untreated cells were used as a control (in grey) and 1 µM condition was also performed after pre-incubation with the selective S1P_2_ antagonist JTE013 (in blue). Histograms represent the mean values ± S.E.M. on 3 independent experiments for each condition, expressed as a percentage of 1 µM S1P response. Statistical significance between maximal activation levels (plateau at 1–2 µM) for different compounds was assessed using one-way ANOVA with a Scheffe’s *post hoc* test: *p < 0.05.

In order to verify that the measured response was not influenced by the coupling system (i.e. co-expression of apoaequorin along with a promiscuous G protein subtype) we then tested if the influence of the alkyl chain length can be observed at the metabolic level. Specifically, we measured NF-kB activation following stimulation of C16, S1P (C18) and C19 compounds analogs in BEAS-2B where such metabolic response to S1P is solely due to endogenous S1P_2_ activation^37^. When we stimulated BEAS-2B cells with various concentrations and measured NF-kB activity (see Experimental Procedures) we observe concentration-dependent response to the S1P and its analogs and the maximal level of activation reached differs from one compound to another (Fig. 4).

The efficacy of the alkyl chain variants are different, with C16 displaying the lowest efficacy, with about 77% of S1P activation, and C19 showing an equal to higher efficacy than S1P (note that E_max_ is not reached at 1 or 2 µM for S1P_2_ in the aequorin assay, see Fig. 3B). The differences between the maximal activation level induced by the different analogs are statistically significant (p < 0.05) (Fig. 4). The trend observed using an S1P_2_-mediated physiological metabolic response is thus consistent with the activation trend deciphered from our aequorin-based assay for S1P_2_ (Fig. 3 and Table 1).

As the differences observed are independent of the functional assay, we can therefore conclude that the alkyl chain is a determinant of receptor activation in terms of G protein-coupling but also of biological outcome.

### Structure-activity relationships at the sphingosine receptor family

**Figure 5:**
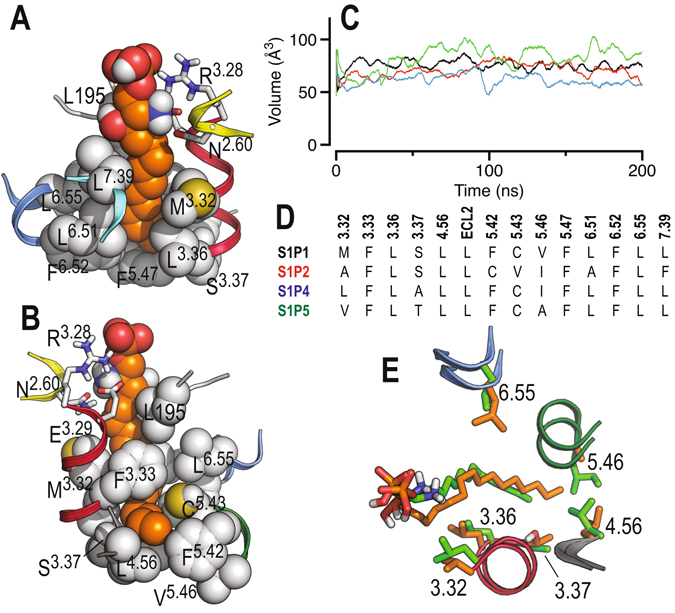
Molecular dynamics simulations of sphingosine-1-phosphate receptors. (**A**) Detailed view of the narrow channel of S1P_1_ where the alkyl tail of S1P (in orange) must expand. The end of the channel is delimited by the amino acids at positions 4.56, 5.42 and 5.46. The structure depicts the final conformation at 0.2 μs. (**B**) Same as in panel A but rotated 180°. (**C**) Volume of the channel in S1P_1_ (black), S1P_2_ (red), S1P_4_ (blue) and S1P_5_ (green) along the MD trajectories with the natural S1P agonist as calculated with POVME^49^. (**D**) Sequence alignment, among sphingosine receptors, of the amino acids forming this channel. (**E**) The final conformation, at 0.2 μs, obtained in the MD simulations of S1P_4_ in complex with C16 (in green) and S1P_5_ in complex with C20 (in orange).

The above results clearly show that ligand chain length influences the activation of sphingosine-1-phosphate receptors in a subtype-dependent manner, with S1P_4_ behaving quite differently. This seems due to the narrow channel (binding cavity), formed by the hydrophobic and bulky side chains of the amino acids at positions 3.32–3.33, 3.36–3.37, 4.56, 5.42–5.43, 5.46–5.47, 6.51–6.52, 6.55 and 7.39, where the alkyl tail must expand (Fig. 5A,B). The end of this channel is delimited by the amino acids at positions 4.56, 5.42 and 5.46, whereas the other side chains define the shape of the channel. We explored the volume of this channel from the conformational ensembles obtained in the MD trajectories of the natural S1P agonist in complex with the different sphingosine-1-phosphate receptor subtypes. Clearly, the amino acid side chains of S1P_4_ delineate the smallest channel, the side chains of S1P_5_ the largest, whereas the channels formed by the side chains of S1P_1_ and S1P_2_ are in between (Fig. 5C). This rank order follows our experimental results in which short alkyl chains (suitable for fitting a small size channel) favor S1P_4_ activation, long alkyl chains (appropriate for fitting a large channel) activate S1P_5_ more efficiently, and S1P_1_ and S1P_2_ favor long alkyl chains but less markedly than S1P_5_. Analysis of the amino acid sequence forming this channel between S1P_4_ (the smallest) and S1P_5_ (the largest) shows differences only at positions 3.32, 3.37 and 5.46 (Fig. 5D). Specifically, S1P_4_ contains the bulky and β-branched side chain of Ile at position 5.46 while S1P_5_ contains the small Ala amino acid at the end of the channel. Notably, mutation of the β-branched V209^5.46^ to Leu in S1P_1_, increasing side chain flexibility due to the lack of the β-branched character of Leu, augments S1P-induced Emax to 147%^14^.

In addition, the side chain at position 3.37 (Ala in S1P_4_ and Thr in S1P_5_) influences the conformation of L^4.56^. In transmembrane α-helices side chain conformations of Thr are mostly limited to *gauche*+^38^ in which the hydroxyl group forms hydrogen bonds with the backbone carbonyl oxygen at position i-3 and the methyl group points toward L^4.56^. The methyl group of Thr forces L^4.56^ to adopt the *gauche*+ conformation pointing away from the bundle and expanding the length of the channel in S1P_5_, in contrast to the small Ala that permits L^4.56^, in the *trans* conformation, to pack against TM 3 making the channel shorter in S1P_4_ (Fig. 5E).

A major question is to understand at the molecular level the different activation trends observed based on E_max_ values, among different ligand chain lengths and receptor subtypes. Thus, we explored the initial structural changes triggered by the C16, C17, C18, C19 and C20 ligands in the S1P_1_, S1P_2_, S1P_4_ and S1P_5_ receptor subtypes by MD simulations (see Experimental Procedures). Although ligand efficacy is a function of multiple factors, we found a clear correlation between E_max_ values and the conformation of L^3.36^ (no significant differences in the side chain conformation of F210^5.47^ are observed), specifically the amount of time the L^3.36^ side chain spends in the inactive *trans* or active *gauche-* conformation (Supplementary Fig. S7). Short alkyl chains (C16-C18) stabilize the active *gauche-* conformation of L^3.36^ better than long alkyl chains (C19-C20) in S1P_4_, whereas this trend is the opposite for S1P_5_ in which long alkyl chains (C19-C20) are necessary for achieving the active conformation in agreement with the experimental rank order of E_max_ obtained for these receptor subtypes (Table 1). In addition, the rank order of E_max_ in S1P_1_ and S1P_2_ (Table 1) also correlates with the amount of time L^3.36^ spends in the active *gauche-* conformation (C16 < C17 ≈ C18 ≈ C19 for S1P_1_ and C16 < C17 < C18 < C19 ≈ C20 for S1P_2_).

## Conclusions

Our study demonstrates that the alkyl chain length of the lipidic agonist is a key feature in activation of S1P receptors. This is achieved by hydrophobic interactions between the lipid tail and residues in a binding pocket located between TMs 3–6. We can also extrapolate these findings to fingolimod-phosphate. The additional methylene units of the fingolimod-phosphate agonist, relative to the ML056 antagonist (Fig. 1), also seems a key feature for their different functional activity. Moreover, subtype-specific effects were observed within S1P receptors family, especially marked in the case of S1P_4_, can be rationalized based on the different volumes of the binding cavities. Our data suggest a model where one region of the ligand (the head group of S1P) is responsible for high-affinity binding, while a distinct part (the alkyl chain) is responsible for triggering activation. Therefore that high affinity binding and receptor activation can be partially uncoupled. This can be illustrated by comparing the profiles of S1P4R, where E_max_ and EC_50_ correlate, with that of S1P2 where it is not the case, as S1P has a better efficacy but a lower potency then C19 or C20.

A key question in GPCR biology is how structurally and chemically diverse ligands can activate receptors with the same overall structure and common signaling effectors, i.e. how the binding of epinephrine on β_2_- and of sphingosine-1-phosphate on S1P_1_ receptor both lead to an outward movement of TM6 at the intracellular side. Obviously, the binding site is specific to the cognate ligand, but what are the conserved structural elements that allow convergence of the conformational pathways? Residues at TM3-5-6 interface interacting with ligand’s alkyl chain have been identified in the activation mechanism of other GPCRs. This suggests the existence of common structural events following agonist-induced structural changes that are conserved throughout class A and where the different conformational pathways converge. Still, these convergence points have required specific adaptation of each receptor to accommodate the structural specificities of their cognate ligand, as illustrated by the lack of the crucial P^5.50^ residue in TM5, a characteristic feature of receptors for lipid mediators.

The model proposed for activation of S1P receptors is likely to be applicable to some extent to the other lipid-binding GPCRs, such as related LPA receptors, but also opens perspectives to understanding the transmission switch in other receptors families. In particular, it would be interesting to investigate whether a dual-site model may apply to other types of GPCRs, as already shown for chemokine receptors^39^.

## Methods

### Molecular dynamics simulations of sphingosine-1-phosphate receptors in complex with ligands

The crystal structure of inactive S1P_1_ (PDB code 3V2Y)^12^ was used for the construction of the S1P_1_ model. The T4-lysozyme used to stabilize the structure of S1P_1_^40^ was removed and ICLs 2 (Met149-Ser155) and 3 (Ser232-Lys243) were modeled using ICL 2 of dopamine D3 (PDB code 3PBL) and ICL 3 of squid rhodopsin (PDB code 2Z73) as templates with Modeller 9.12^41^. The natural S1P agonist was docked into the S1P_1_ receptor using the Autodock Vina tool^42^. All docking solutions were visually inspected and the poses in which the zwitterionic head-group interacts with N101^2.60^, R120^3.28^ and E121^3.29^ in a similar way as the co-crystalized ML056 antagonist were energy minimized. In these docking poses the alkyl chain of S1P expands towards TMs 3, 5 and 6, similarly to previous proposed docking models^12, 20^. An alternative conformation of S1P in which the alkyl chain is pointing towards the intracellular part was also considered (Fig. 2A). In parallel, an “active” conformation of S1P_1_ was modeled from the crystal structure of a nanobody-stabilized active state of the β_2_-adrenergic receptor (PDB id 3P0G)^23^, by changing the conformation of the intracellular part of the TM helices of S1P_1_ (Ala130^3.38^ – Trp168^4.50^, Ser216^5.53^ – Ile266^6.45^, Ser304^7.46^ – Leu330^CTerm^) for the active conformation of β2- (Ala119^3.38^ – Trp158^4.50^, Ile214^5.53^ – Thr283^6.45^, Ser319^7.46^ – Leu342^CTerm^) (see Supplementary Fig. S2 for details). Modeller 9.12 was used to build homology models of “active” S1P2 (33% of sequence identity and 47% of sequence similarity), S1P4 (26% and 41%) and S1P5 (29% and 43%) subtypes using the constructed structure of “active” S1P_1_ as a template. These “active-like” models include the G-protein-mimetic nanobody^23^. Autodock Vina was also used to dock C16, C17, C18 (S1P), C19, and C20 ligands into the different “active” models of receptor subtypes. For comparison purposes the crystal structures of the inactive, carazolol-bound, β_2_-adrenergic receptor (PDB id 2RH1)^43^ and the nanobody-stabilized active β_2_-adrenergic receptor bound to the BI167107 agonist (PDB id 3P0G)^23^ were also studied. VMD and its membrane and solvate plugins were used to embed these computational models into a constructed POPC bilayer^44^. All molecules closer than 2 Å to any receptor atom were removed from the system. The resulting models, which consist in a rectangular box containing a lipid bilayer (~170 molecules of POPC) with explicit solvent (>16,000 water molecules) and a 0.15 M concentration of Na^+^ and Cl^−^ ions (~120 and 140 ions, respectively), were energy-minimized and subsequently subjected to a 10 ns MD equilibration with positional restraints on protein coordinates to remove possible voids present in protein/lipids or proteins/water interfaces. After 10 ns, these restraints were released and MD trajectories were produced at constant pressure and temperature: 2 μs for inactive S1P_1_ + C18 ligand (S1P), two different replicas of 0.5 μs for “active” S1P_1_ + C18 ligand + nanobody, 0.5 μs for “active” S1P_1_+ no ligand + nanobody, 0.2 μs for “active” receptor subtypes (S1P_1_-S1P_5_) with each of the ligands (compounds C16-C20) + nanobody, 0.5 μs for inactive β_2_- + carazolol and 0.5 μs for active β_2_- + BI167107 + nanobody (see Supplementary Table S1). Computer simulations were performed with the GROMACS 4.6 simulation package^45^, using the AMBER99SB-ILDN force field as implemented in GROMACS, Berger parameters for POPC lipids, and the general Amber force field (GAFF) and HF/6-31 G*-derived RESP atomic charges for the ligands. This procedure has been previously validated^46^.

### Chemistry

Full synthetic details as well as characterization data of final compounds **1**–**4**, together with synthesis of all intermediates, are described in the Supporting Information.

### Functional characterization of S1P analogs at S1P receptors

#### Cell lines

All cell culture work on S1P receptors was performed using AequoScreen^®^ CHO-K1 cell lines provided by Perkin Elmer, which are modified to stably express the mitochondrially targeted apo-aequorin protein (transfection with a bicistronic expression plasmid). CHO-K1 Parental Aequorin cell line (No. ES-000-A24) is stably expressing the mitochondrially targeted apoaequorin and the promiscuous G protein subtype Gα1, redirecting the coupling towards the PLC-calcium signaling cascade. This cell line was used for activation assays on constitutively expressed S1P_2_ (EDG5) receptor as well as for transient transfection and activation assay on human S1P_1_ (EDG1) receptor (see below). Activation assays on human S1P_4_ and S1P_5_ receptors were performed using aequorin CHO-K1 cells lines stably expressing S1P_4_ (ref No. ES-592-A) and S1P_5_ (ref No. ES-593-A) respectively, as well as the apoaequorin and the promiscuous Gαq/i(5) protein.

S1P_2_ functional response was assessed using the parental cell line (CHO-Aeq) and S1P_5_ cell line. Indeed, CHO-Aeq cells display significant S1P activity that is solely due to S1P_2_ as we could fully inhibit it using the S1P_2_ specific antagonist JTE013 (Supplementary Fig. S5). S1P_4_ expression was adequate for our assays. In contrast, S1P_5_ levels were low but experiments performed in presence of the specific S1P_2_ antagonist JTE013 to inhibit endogenous S1P_2_ signal which allowed to properly characterize S1P_5_ response.

#### Cell culture

All AequoScreen^®^ CHO-K1 cell lines were grown at 37 °C and 5% CO_2_, in HAM’s F12 Nutrient Mix medium (Gibco, Life Technologies), supplemented with 10% fetal bovine serum (Biowest), penicillin and streptomycin at 50 units/mL (BioWhittaker, Lonza), and 1 mM sodium pyruvate (BioWhittaker, Lonza). Depending on the cell line, the following selection antibiotics were added to the media: 500 μg/mL Geneticin^®^ (G418 sulfate) (Gibco, Life technologies) for the S1P receptors expression selection, 250 μg/mL Zeocin (Invitrogen, Life technologies) for the apoaequorin expression selection, 5 μg/mL Puromycin (Invitrogen, Life technologies) for promiscuous Gαq/i(5) protein expression selection. Cells were passaged, after one wash with Ca^2+^ and Mg^2+^-free Dubelcco’s Phosphate Saline Buffer (PBS), using Trypsin-EDTA mixture at 170 U/mL–0.2 mg/mL (BioWhittaker, Lonza).

#### Transient transfection for S1P_1_ expression

AequoScreen^®^ CHO-K1 parental cell line stably transfected with pCAEQG plasmid leading to the co-expression of apoaequorin along with the promiscuous G protein subtype Gα16 were grown as described in section B, except from the replacement of regular FBS by Ultra-low Endotoxin FBS (BioWest). CHO-K1 parental cells were seeded in 6-well pates (Greiner) in medium without antibiotics 24 h before transfection, to reach a 50–70% confluency. Transient transfection with a pcDNA3.1+ vector encoding the human gene *S1PR1* with an N-terminal HA-tag sequence (Missouri S&T cDNA Resource center), purified with an EndoFree Plasmid Maxi kit (Qiagen). Transient transfection was performed using the cationic polymer JetPei^®^ (Polyplus-transfection) transfection agent at a 2:1 agent:DNA ratio (4 μg DNA/well), that gave the best expression based on western-blotting analysis and S1P functional response. After removal of the transfection agent cells were subjected to a 12 days antibiotic selection (G418, Life technologies) before performing the intracellular calcium mobilization assays.

#### Aequorin-based calcium mobilization assay

Functional response was assessed by measuring the luminescence of mitochondrial aequorin after agonist-induced intracellular Ca^2+^ release^47^. Antibiotics were removed from the cell medium 24 hours before the assays. Cells at 70–80% confluency were collected from culture plates with Ca^2+^ and Mg^2+^-free Dulbecco’s phosphate-buffered saline (Lonza) containing 1 mM EDTA (Invitrogen, Life Technologies), centrifuged at 1000 g for 5 min and resuspended at a density of 5.10^6^ cells/mL in Dulbecco’s modified Eagle’s medium (DMEM) w/o phenol red supplemented with 0, 1% BSA (Sigma). Cells were incubated for 4 hours in the dark in the presence of the Aequorin chromophore coelenterazine H (Promega) at 5 μM. Cells were diluted 10 times in DMEM - 0, 1% BSA prior to use. 25000 cells (50 μL) were injected to each well of white 96-well plates Lumitrac200 (Greiner bio-one) containing the prepared dilutions of the ligands (50 μL) in DMEM-0, 1% BSA, ranging from 0.1 nM to 30 μM final concentration. When indicated, specific S1P_2_ antagonist JTE013 was added at a concentration corresponding to 20 times the IC_50_ and incubated for 15 min before performing the measurements. For inhibition curve determination (“antagonist mode”), cells were pre-incubated in wells with increasing concentrations of antagonist for 15 min and luminescence is measured after addition of S1P at the EC_80_ *(concentration at 80% of the effect)*. The emitted light was measured for 30 sec with a CentroXS3 LB960 Luminometer (Berthold Technologies). Analysis of kinetics indicates that the luminescence signal was always contained within the 30 second windows independently of the analog or receptor tested (not shown). Duplicate measurements were performed for each concentration and every experiment with analogs was performed between 2 and 4 times depending on the subtype. As a positive control ATP (Sigma) at 20 μM was used, which leads to full activation of the endogenous purinergic receptors, allowing signal normalization for agonist response. Data were analyzed with the PRISM3.0 software (Graph-Pad Prism Software, San Diego, CA) using nonlinear regression fittings with sigmoidal dose-response model parameters, to determine EC_50_ *(half maximal effective concentration)* or IC_50_ *(half maximal inhibitory concentration)* and E_max_ *(maximal efficacy)* values. To compare the different S1P analogs, E_max_ values were further normalized on maximal response to the cognate agonist, S1P.

#### NF-κB luciferase reporter assay

Human BEAS-2B NF-κB luciferase reporter cells (provided by Dr. Simon Rousseau, McGill University) were seeded into 24-well plates at a density of 50000 cells/well and grown for 24 hours in DMEM (Lonza) supplemented with 10% fetal bovine serum (Biowest), penicillin and streptomycin at 50 units/mL (BioWhittaker, Lonza) and hygromycin B selection antibiotic (Invivogen) at 100 µg/mL. Cells were serum-starved overnight in DMEM supplemented with 0.1% bovine serum albumin (Sigma) and stimulated for 4 hours at 37 °C with C16, S1P or C19, ranging from 10 nM to 2 µM final concentration. Other cells were pretreated with the S1P_2_ antagonist JTE013 at 3 µM and then stimulated with 1 µM of C16, S1P or C19. At the end of the incubation with the compounds, cells were lysed with Reporter Lysis Buffer (Promega), the lysates were collected and centrifuged at 12000 g, 4 °C for 3 min. 10 µL of each supernatant were used for luciferase activity measurement. Luminescence intensity after addition of 40 µL of D-luciferin (Biosynth) reagent solution at 235 µM was measured for 15 sec with a CentroXS3 LB960 Luminometer (Berthold Technologies). Results were normalized on supernatants total protein content and expressed as a percentage of 1 µM S1P response. Dose response-curves were limited to the 10 nM–2 µM range because we observed irregular or non-reproducible data for values above 2 µM. Such erratic behavior is most likely due to toxic effects caused by long exposure (4 h) to high concentration of S1P (and analogs) which have been previously described can be receptor independent^48^. For this concentration range, according to our calcium mobilization assays, E_max_ should be reached for C16, barely for S1P and not yet for C19 (Fig. 3).

#### Statistical analysis

Data were fitted into a sigmoidal concentration-response curve using the GraphPad Prism software (San Diego, California, US). Statistical analysis was performed using one-way ANOVA with Scheffe’s post-test.

## Electronic supplementary material

Supplementary Information
