# Supplementary material for: Ligand chain length drives activation of lipid G protein-coupled receptors
Source: Sci Rep. 2017 May 17;7:2020. doi: 10.1038/s41598-017-02104-5 (PMC5435731; doi:10.1038/s41598-017-02104-5)
Supplement: Supplementary file 1 — Supplementary Information [file 41598_2017_2104_MOESM1_ESM.pdf]

Ligand chain length drives activation of lipid G protein-coupled receptors

Anastassia Troupiotis-Tsaïlaki[1*], Julian Zachmann[2*], Inés González-Gil[3], Angel Gonzalez[2], Silvia Ortega-Gutiérrez[3], Maria L. López-Rodríguez[3], Leonardo Pardo[2&] and Cedric Govaerts[1&]

[1]Laboratoire de Structure et Fonction des Membranes Biologiques, Université Libre de Bruxelles, Brussels, Belgium

[2]Laboratori de Medicina Computacional, Unitat de Bioestadística, Facultat de Medicina, Universitat Autònoma de Barcelona, 08193 Bellaterra, Barcelona, Spain

[3]Departamento de Química Orgánica I, Facultad de Ciencias Químicas, Universidad Complutense de Madrid, E-28040 Madrid, Spain

[*]These authors contributed equally

Correspondence: Cedric.Govaerts@ulb.ac.be & Leonardo.Pardo@uab.cat

Lead contact : Cedric Govaerts.

SUPPLEMENTARY INFORMATION

1. Supplementary Figures

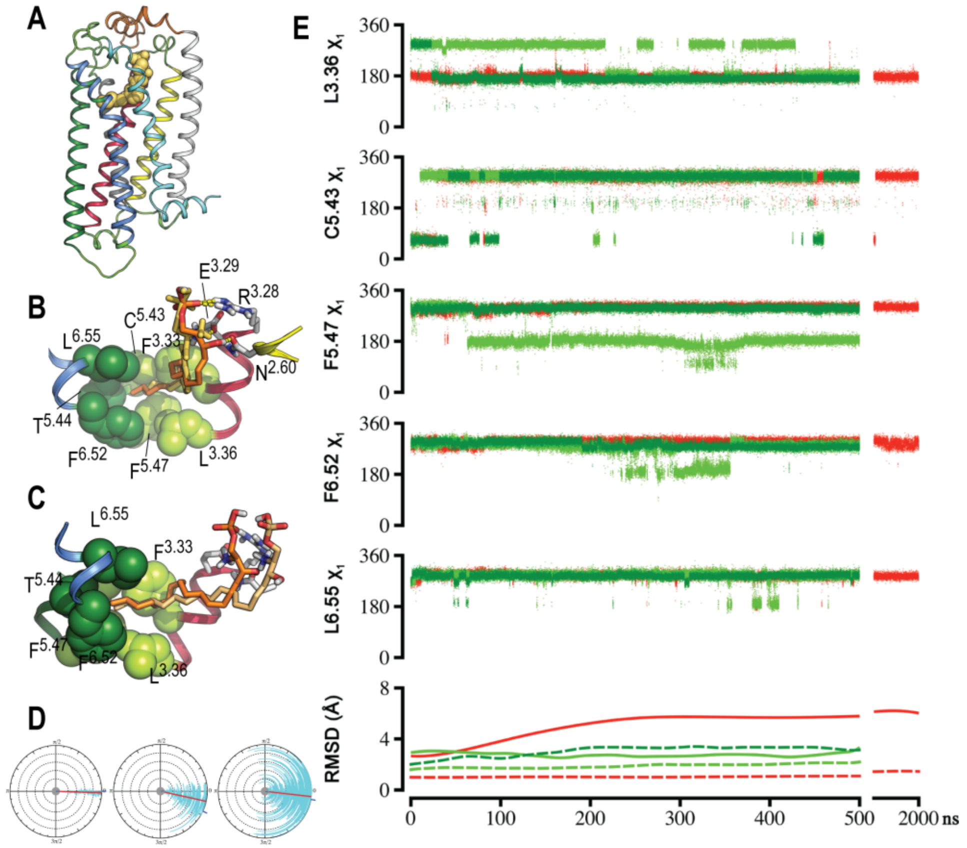

Figure S1. **Molecular dynamics simulations of S1P$_1$ in complex with the natural S1P agonist**

- **A.** Crystal structure of S1P$_1$ in complex with the co-crystalized ML056 antagonist (yellow spheres).
- **B.** Molecular model of the natural S1P agonist (in orange) docked into the inactive crystal structure of S1P$_1$. The ML056 antagonist (in yellow) is shown as a reference. The terminal part of the alkyl chain of S1P points toward bulky amino acids located in either TMs 3 and 5 (shown as light green van der Waals spheres) or TMs 5 and 6 (dark green spheres).
- **C.** The final conformation, at 0.5 μs, of S1P (in light orange) in complex with the inactive conformation of S1P$_1$ obtained during the MD simulation. The initial conformation at 0 μs of S1P (in orange) is shown as a reference.
- **D.** Dial plots of the rotation of TM 3 from position 3.25 to position 3.40 (cyan line), around the helix axis (calculated from the Cα atoms), during the MD simulations of inactive- (left panel), active- (central panel), and ligand-free (right panel) models of S1P$_1$ as calculated with the Trajelix module of Simulaid[1]. The radius is the simulation time (0.5 μs). The line in the central disk gives the initial value, the red line gives the average value and the blue tick outside the disc shows the final value. The small fluctuation in inactive receptor (top panel) is due to the packing of TM 3 with TM 5 (see Supplementary Fig. S1B), the medium fluctuation of the active receptor (central panel) is due to the fact that active states of GPCRs are significantly more dynamic than inactive ones[2], and the large fluctuation of the bottom panel is attributed to the lack of ligand so that TM 3 is freer to move than in the other MD simulations.
- **E.** Evolution of dihedral $\chi 1$ angles of L128$^{3.36}$, C206$^{5.43}$, F210$^{5.47}$, F273$^{6.52}$ and L276$^{6.55}$ side chains, and RMSD values of the zwitterionic head-group of S1P (solid line) and the Cα atoms of S1P$_1$ (dashed line), relative to the initial structure, obtained during the MD simulations of inactive- (in red) and active- (in light and dark green) like conformation of S1P$_1$ in complex with S1P (light green) and ligand-free (dark green) (see Methods). Two independent replicas of 0.5 μs of the active-like model in complex with S1P$_1$ were performed (only one of them is shown for clarity).

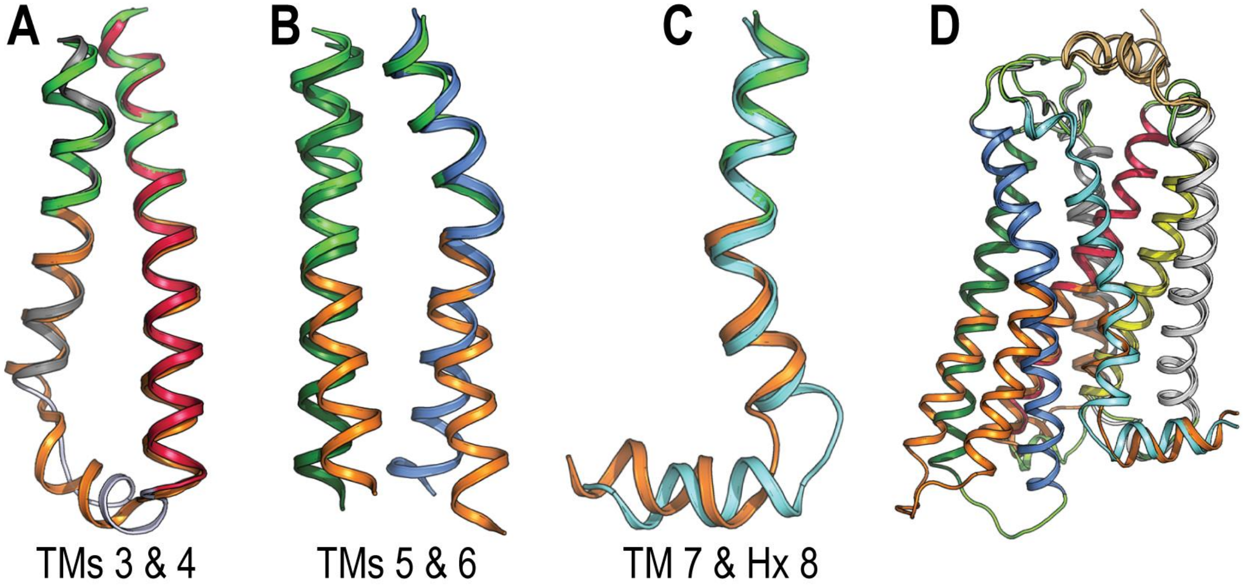

Figure S2. Construction of the "active" conformation of S1P$_1$ from the crystal structure of a nanobody-stabilized active state of the β_2-adrenergic receptor

A. TMs 3 & 4. The extracellular part Cys106$^{3.25}$ – Ala119$^{3.38}$ and Trp158$^{4.50}$ – Gln170$^{4.62}$ of "active" β_2– (shown in light green) (PDB id 3P0G) was superimposed to Trp117$^{3.25}$ – Ala130$^{3.38}$ and Trp168$^{4.50}$ – Met180$^{4.62}$ of "inactive" S1P$_1$ (red and grey) (PDB id 3V2Y). The "active" conformation of TMs 3 & 4 of S1P$_1$ were modeled by changing the conformation of the intracellular Ala130$^{3.38}$ – Trp168$^{4.50}$ of S1P$_1$ (red and grey) by the conformation of Ala119$^{3.38}$ – Trp158$^{4.50}$ of "active" β_2- (orange).

B. TMs 5 & 6. The extracellular part Tyr199$^{5.38}$ – Ile214$^{5.53}$ and Thr283$^{6.45}$ – His296$^{6.58}$ of "active" β_2– (light green) was superimposed to His201$^{5.38}$ – Ser216$^{5.53}$ and Ile266$^{6.45}$ – Asp279$^{6.58}$ of "inactive" S1P$_1$ (dark green and blue). The "active" conformation of TMs 5 & 6 of S1P$_1$ were modeled by changing the conformation of the intracellular Ser216$^{5.53}$ – Ile266$^{6.45}$ of S1P$_1$ (dark green and blue) by the conformation of Ile214$^{5.53}$ – Thr283$^{6.45}$ of "active" β_2- (orange).

C. TM 7 & Hx8. Ile309$^{7.36}$ – Ser319$^{7.46}$ of "active" β_2– (light green) was superimposed to Glu294$^{7.36}$ – Ser304$^{7.46}$ of "inactive" S1P$_1$ (cyan). The "active" conformation of TM 7 & Hx8 of S1P$_1$ were modeled by changing the conformation of the intracellular Ser304$^{7.46}$ – Leu330^{CTerm} of S1P$_1$ (cyan) by the conformation of Ser319$^{7.46}$ – Leu342^{CTerm} of "active" β_2- (orange).

D. Backbone superimposition of "inactive" and "active" S1P$_1$. The color code is TM 1 in white, TM 2 in yellow, TM 3 in red, TM 4 in grey, TM 5 in dark green, TM 6 in blue, and TM 7 in cyan. The part of "active" S1P$_1$ that was modeled from β_2– is shown in orange. The extracellular part of the active-like model of S1P$_1$ is identical to the inactive model.

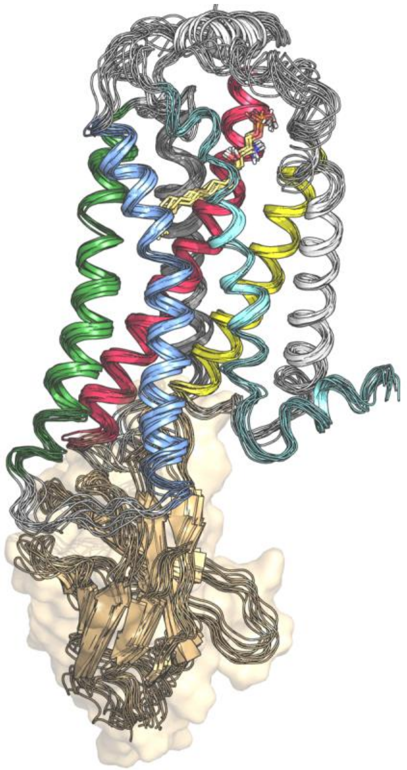

Figure S3. MD simulations of the "active" conformation of S1P$_1$ in complex with the natural S1P agonist and the G protein-mimetic nanobody.

Cartoon representation of twenty snapshots extracted from 500 ns MD trajectory. The color code of the helices is as in Figure S2, the S1P agonist is shown in yellow, and the nanobody in light orange (transparent surface). Root mean-square deviations (RMSD) of the simulation are shown in Figure S1D.

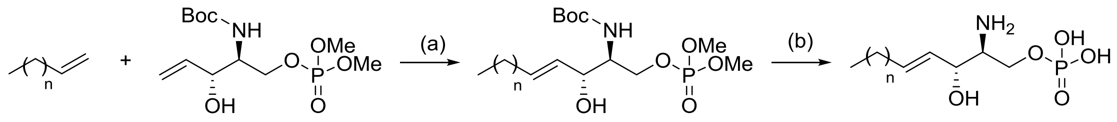

n = 10, 11, 13, 14

5

6: n = 10
7: n = 11
8: n = 13
9: n = 14

1: n = 10
2: n = 11
3: n = 13
4: n = 14

Figure S4. Synthesis of S1P derivatives of variable chain length

Synthetic scheme. Reagents and conditions: (a) Grubbs cat. 2nd, DCM, reflux, 2 h, 20-25%; (b) i) TMSBr, DCM, rt, 4 h, ii) CH_3OH, rt, 10 min, 99% (2 steps).

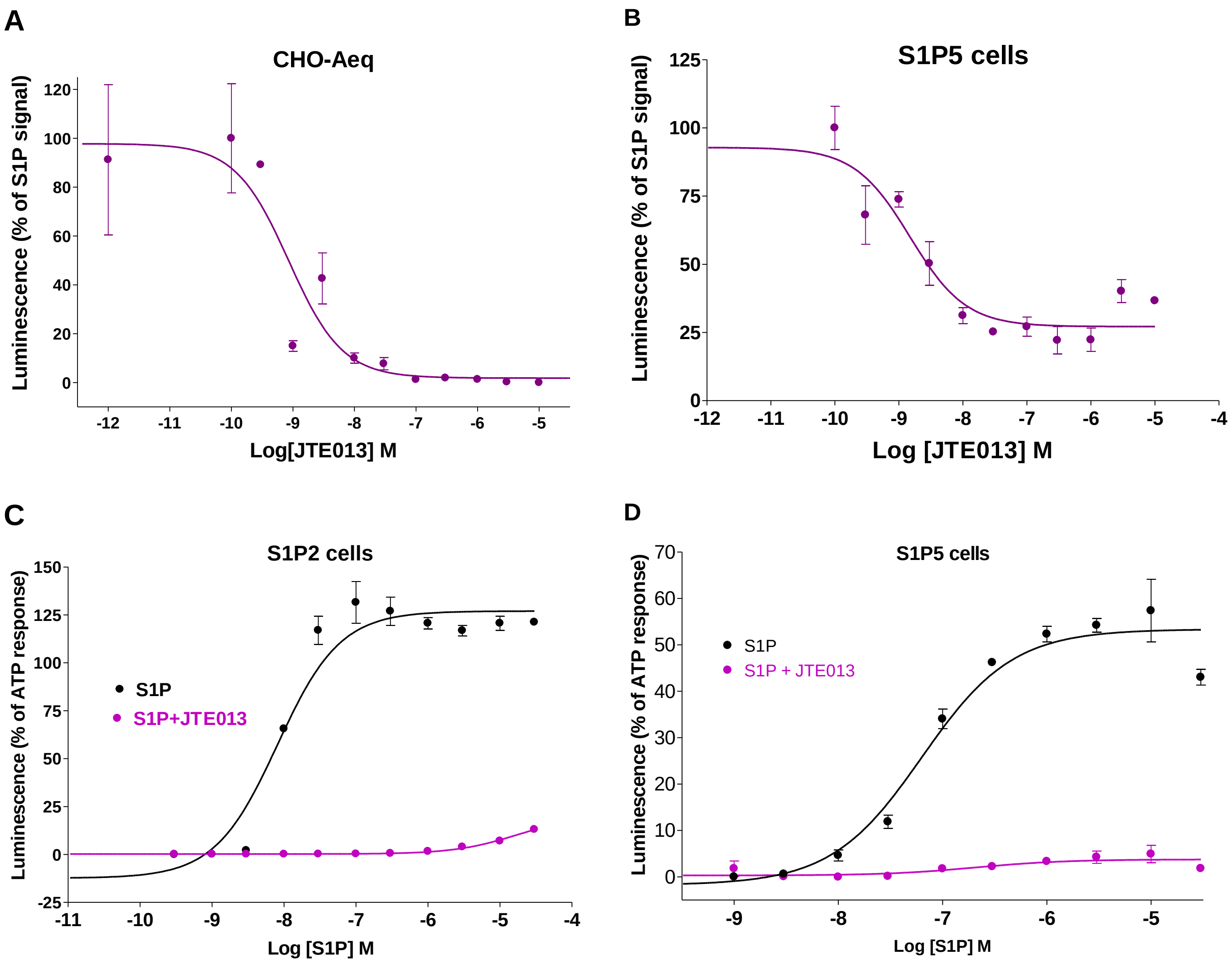

Figure S5. Inhibition of $S1P_2$ response to S1P by the specific antagonist JTE013

A/ CHO-Aeq cells pre-incubated with increasing concentrations of the $S1P_2$-specific antagonist JTE013 were stimulated with 1µM of S1P and the resulting luminescence was measured. Data points plotted represent the mean ± S.E.M of duplicates and are expressed as a % of response to S1P 1µM without antagonist. B/ Similar experiment performed on stable $S1P_5$-CHO-Aeq cells. C/ Stable $S1P_2$-CHO-Aeq cells were stimulated by increasing concentrations of S1P in the presence (purple curve) or absence (black curve) of a saturating concentration of JTE013 (20 times the IC_{50}). Data points plotted represent the mean ± S.E.M of duplicates and are normalized on the response to 20 µM ATP (full purinergic receptors activation). D/ Similar experiment performed on stable $S1P_5$-CHO-Aeq cells.

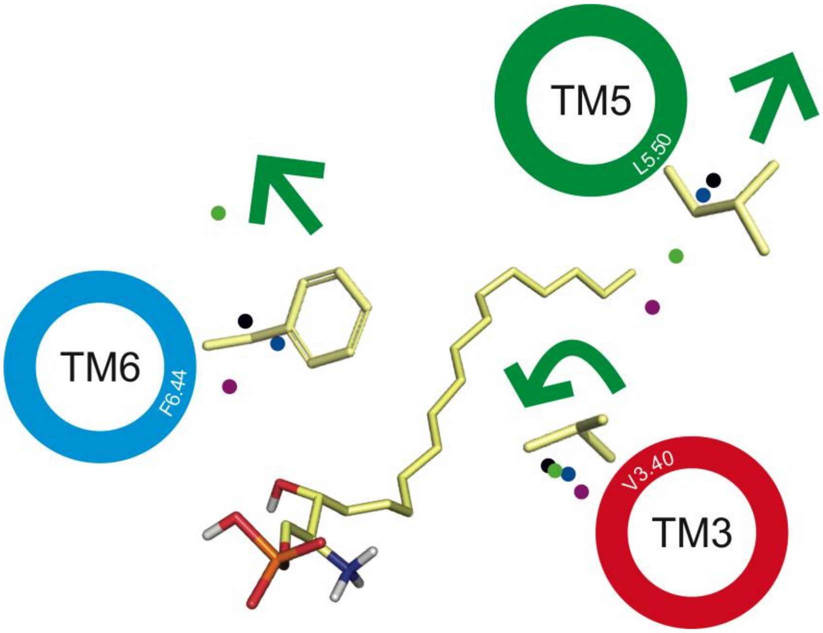

Figure S6. Mechanism of activation of $S1P_1$ by ligands with different chain length

Plot of the centroid (calculated from 200 snapshots) of the Cβ atoms of $V132^{3.40}$, $L213^{5.50}$ and $F265^{6.44}$ ('transmission switch') obtained during 200 ns of MD simulations of $S1P_1$ receptor bound to C16 (purple), C17 (blue), C18/S1P (black), and C19 (green). Arrows show the proposed mechanism of $S1P_1$ activation in which an outward movement of TM 5, an anticlockwise rotation of TM 3, and an outward movement of TM 6 occur. The rank order of E_{max} observed for the different ligands (Table 1) correlates with these movements: C16 (E_{max} 78%) < C17 (107%) ≈ C18 (100%) ≈ C19 (116%). Similar analysis for the other receptor subtypes could not be performed because their homology models displayed increased fluctuations in the MD simulations relative to the $S1P_1$ structure, as revealed by their Cα root-mean-square deviations, that complicates this subtle analysis of helices movements. The position of the helices, the side chains and S1P are shown for clarity.

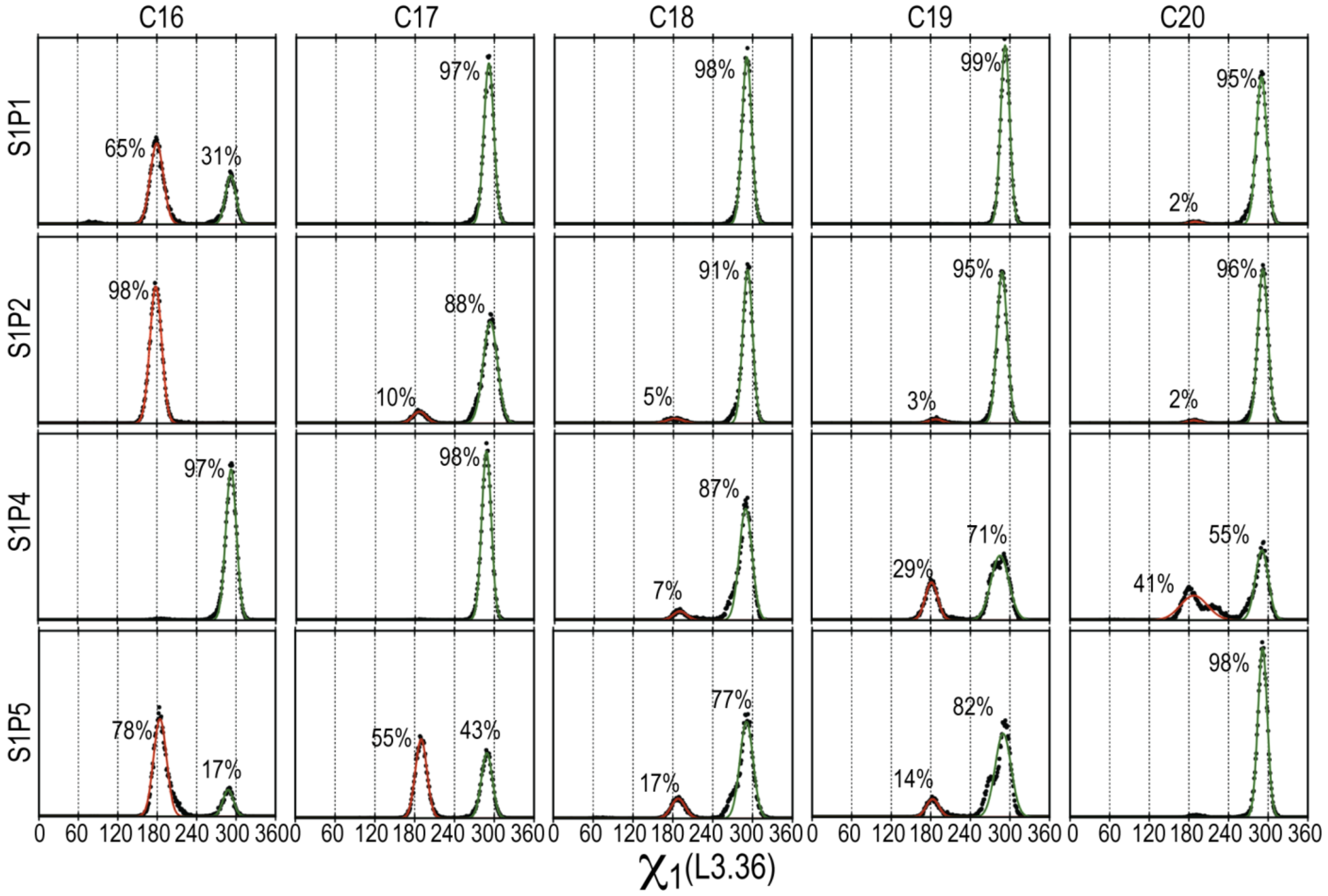

Figure. S7. Structure-activity relationships at the sphingosine receptor family

Radial distribution function for the $\chi 1$ dihedral angle of $L^{3.36}$ (black spheres) obtained in 200ns MD simulations of the C16, C17, C18, C19, and C20 ligands in complex with the sphingosine $S1P_1$, $S1P_2$, $S1P_4$ and $S1P_5$ subtypes. These values were non-linear curve fitting to two Gaussian functions using the MagicPlot software. The area of each Gaussian function, representing the amount of time the $L^{3.36}$ side chain is in the inactive *trans* (in red) or active *gauche-* (in green) conformation, is shown.

Supplementary Tables

Table S1. Summary of the MD simulations performed in this work.

**Receptor**	**Ligand**	**nanobody**	**time**
Inactive $S1P_1$	C18 (S1P)	no	2 μs
“Active” $S1P_1$	C18 (S1P) “up”	yes	0.5 μs
“Active” $S1P_1$	C18 (S1P) “down”	yes	0.5 μs
“Active” $S1P_1$		yes	0.5 μs
Inactive β_2-R	carazolol	no	0.5 μs
Active β_2-R	BI167107	yes	0.5 μs
“Active” $S1P_1$	C16	yes	0.2 μs
“Active” $S1P_1$	C17	yes	0.2 μs
“Active” $S1P_1$	C19	yes	0.2 μs
“Active” $S1P_1$	C20	yes	0.2 μs
“Active” $S1P_2$	C16	yes	0.2 μs
“Active” $S1P_2$	C17	yes	0.2 μs
“Active” $S1P_2$	C18 (S1P)	yes	0.2 μs
“Active” $S1P_2$	C19	yes	0.2 μs
“Active” $S1P_2$	C20	yes	0.2 μs
“Active” $S1P_4$	C16	yes	0.2 μs
“Active” $S1P_4$	C17	yes	0.2 μs
“Active” $S1P_4$	C18 (S1P)	yes	0.2 μs
“Active” $S1P_4$	C19	yes	0.2 μs
“Active” $S1P_4$	C20	yes	0.2 μs
“Active” $S1P_5$	C16	yes	0.2 μs
“Active” $S1P_5$	C17	yes	0.2 μs
“Active” $S1P_5$	C18 (S1P)	yes	0.2 μs
“Active” $S1P_5$	C19	yes	0.2 μs
“Active” $S1P_5$	C20	yes	0.2 μs
		**Total**	**8.3 μs**

Supplementary Experimental Procedures

Unless otherwise stated, the starting materials, reagents, and solvents used were high-grade commercial products from Sigma-Aldrich, ABCR, Lancaster, Scharlab, or Panreac. Dichloromethane (DCM) was dried using a Pure Solv^{TM} Micro 100 Liter solvent purification system. All reactions were performed under an argon atmosphere in oven-dried glassware. Analytical thin-layer chromatography (TLC) was run on Merck silica gel plates (Kieselgel 60 F-254), with detection by UV light (λ = 254 nm) or 10% phosphomolybdic acid solution in ethanol. Flash chromatography was performed on a Varian 971-FP flash purification system using silica gel cartridges (Varian, particle size 50 µm). All compounds were obtained as oils. Infrared (IR) spectra were measured on a Bruker Tensor 27 instrument equipped with a Specac ATR accessory of 5200-650 cm^{-1} transmission range; frequencies (ν) are expressed in cm^{-1}. Optical rotation [α] was measured on a Perkin Elmer 241 polarimeter using a sodium lamp (λ = 589 nm) with a 1 dm path length; concentrations are given as g/100 mL. ^{1}H, ^{13}C and ^{31}P Nuclear Magnetic Resonance (NMR) spectra were recorded on a Bruker Avance 500MHz (^{1}H, 500 MHz; ^{13}C, 125 MHz, ^{31}P, 202 MHz) or Bruker DPX 300MHz (^{1}H, 300 MHz; ^{13}C, 75 MHz) instrument at room temperature at the Universidad Complutense de Madrid (UCM)'s NMR core facility. Chemical shifts (δ) are expressed in parts per million relative to internal tetramethylsilane; coupling constants (*J*) are in hertz (Hz). The following abbreviations are used to describe peak patterns when appropriate: s (singlet), d (doublet), t (triplet), q (quartet), qt (quintuplet), m (multiplet), dd (double doublet), ddd (double double doublet), dt (double triplet), br (broad), and app (apparent). High resolution mass spectrometry (HRMS) was carried out on a FTMS Bruker APEX Q IV spectrometer in electrospray ionization (ESI) at UCM's mass spectrometry core facility. For all final compounds, purity was determined by high-performance liquid chromatography coupled to mass spectrometry (HPLC-MS), and satisfactory chromatograms confirmed a purity of at least 95% for all tested compounds. HPLC-MS analysis was performed using an Agilent 1200LC-MSD VL instrument. LC separation was achieved with a Zorbax Eclipse XDB-C18 column (5 µm, 4.6 mm x 150 mm) together with a guard column (5 µm, 4.6 mm x 12.5 mm). The gradient mobile phases consisted of A (95:5 water/methanol) and B (5:95 water/methanol) with 0.1% ammonium hydroxide and 0.1% formic acid as the solvent modifiers. MS analysis was performed with an ESI source. The capillary voltage was set to 3.0 kV and the fragmentor voltage was set at 25 eV. The drying gas temperature was 350 ºC, the drying gas flow was 10 L/min, and the nebulizer pressure was 20 psi. Spectra were acquired in positive or negative ionization mode from 100 to 1000 m/z and in UV-mode at four different wavelengths (210, 230, 254, and 280 nm).

2-[(*tert*-Butoxycarbonyl)amino]-2,4,5-trideoxy-1-*O*-(dimethoxyphosphoryl)-D-*erythro*-pent-4-enitol (**5**) was synthesized as previously described[3] and its spectroscopic data were consistent with those reported[3].

1.1. Synthesis of Compounds 1-4

General procedure for the synthesis of intermediates 6-9

To a solution of **5** (1 equiv) in anhydrous DCM (15 mL/mmol) in a sealed tube, the corresponding terminal alkene (4 equiv) and Grubbs catalyst 2nd generation (0.03 equiv) were added at room temperature. The reaction mixture was refluxed for 2 h. Afterward, the solvent was removed under reduced pressure and the crude was purified by flash chromatography (DCM to DCM/ethyl acetate, 4:1) to afford compounds **6-9**.

***tert*-Butyl ((1*S*,2*R*,3*E*)-1-{[(dimethoxyphosphoryl)oxy]methyl}-2-hydroxypentadec-3-en-1-yl)carbamate (6)**

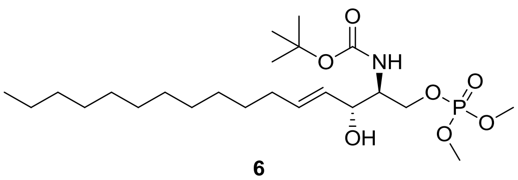

6

Obtained from 1-tridecene (134 mg, 0.74 mmol) and **5** (60 mg, 0.18 mmol) in 25% yield. R_f: 0.21 (DCM/ethyl acetate, 1:1). ^{1}H-NMR ($CDCl_3$, 300 MHz): δ 0.88 (t, *J* = 6.7, 3H, CH_3), 1.21-1.38 (m, 18H, 9CH_2), 1.43 (s, 9H, 3CH_3), 1.98 (br s, 1H, OH),

2.03 (app q, *J* = 6.6, 2H, $C\underline{H}_2CH_{alkene}$), 3.78 (d, *J* = 11.1, 3H, OCH_3), 3.79 (d, *J* = 11.1, 3H, OCH_3), 3.78-3.82 (m, 1H, $C\underline{H}NH$), 4.08-4.16 (m, 2H, CH_2O), 4.33 (ddd, *J* = 10.7, 7.8, 4.7, 1H, $C\underline{H}OH$), 5.01 (br d, *J* = 8.3, 1H, NH), 5.50 (dd, *J* = 15.4, 7.0, 1H, $C\underline{H}_{alkene}CH$), 5.75 (dt, *J* = 15.4, 6.3, 1H, $CH_2C\underline{H}_{alkene}$). ^{13}C-NMR ($CDCl_3$, 75 MHz): δ 14.2 ($CH_3$), 22.8 ($CH_2$), 28.5 ($3CH_3$), 29.2, 29.4, 29.5, 29.6, 29.7, 29.8, 29.81, 32.0, 32.4 ($9CH_2$), 54.6 (d, *J* = 6.0, $2OCH_3$), 55.1 (d, *J* = 3.4, CHNH), 66.8 (d, *J* = 5.8, CH_2O), 72.6 (br s, CHOH), 79.9 (C), 128.6, 135.0 ($2CH_{alkene}$), 155.8 (CO).

***tert*-Butyl ((1*S*,2*R*,3*E*)-1-{[(dimethoxyphosphoryl)oxy]methyl}-2-hydroxyhexadec-3-en-1-yl)carbamate (7)**

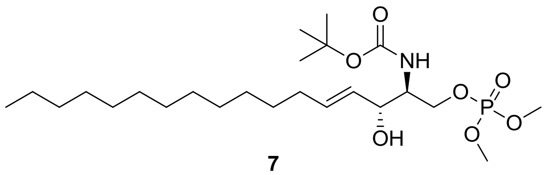

7

Obtained from 1-tetradecene (123 mg, 0.63 mmol) and **5** (51 mg, 0.16 mmol) in 20% yield. R_f: 0.25 (DCM/ethyl acetate, 1:1). ^{1}H-NMR ($CDCl_3$, 300 MHz): δ 0.87 (t, *J* = 6.7, 3H, CH_3), 1.25-1.39 (m, 20H, $10CH_2$), 1.43 (s, 9H, $3CH_3$), 2.03 (app q, *J* = 6.9, 2H, $C\underline{H}_2CH_{alkene}$), 2.25 (br s, 1H, OH), 3.78 (d, *J* = 11.1, 3H, OCH_3), 3.79 (d, *J* = 11.1, 3H, OCH_3), 3.76-3.82 (m, 1H, $C\underline{H}NH$), 4.08-4.15 (m, 2H, CH_2O), 4.33 (ddd, *J* = 10.7, 7.8, 4.8, 1H, $C\underline{H}OH$), 5.03 (br d, *J* = 8.1, NH), 5.49 (dd, *J* = 15.4, 7.0, 1H, $C\underline{H}_{alkene}CH$), 5.75 (dt, *J* = 15.4, 6.6, 1H, $CH_2C\underline{H}_{alkene}$). ^{13}C-NMR ($CDCl_3$, 75 MHz): δ 14.2 ($CH_3$), 22.8 ($CH_2$), 28.5 ($3CH_3$), 29.2, 29.4, 29.5, 29.6, 29.7, 29.8 ($6CH_2$), 29.81 ($2CH_2$), 32.1, 32.5 ($2CH_2$), 54.7 (d, *J* = 6.0, $2OCH_3$), 55.0 (d, *J* = 3.0, CHNH), 66.8 (d, *J* = 5.9, CH_2O), 72.6 (br s, CHOH), 79.9 (C), 128.6, 135.1 ($2CH_{alkene}$), 152.5 (CO).

***tert*-Butyl ((1*S*,2*R*,3*E*)-1-{[(dimethoxyphosphoryl)oxy]methyl}-2-hydroxyoctadec-3-en-1-yl)carbamate (8)**

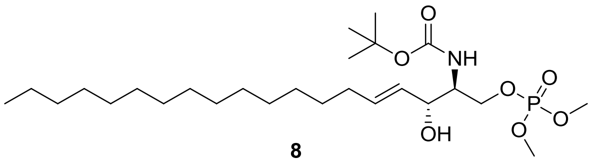

8

Obtained from 1-hexadecene (117 mg, 0.52 mmol) and **5** (42 mg, 0.13 mmol) in 24% yield. R_f: 0.29 (DCM/ethyl acetate, 1:1). ^{1}H-NMR ($CDCl_3$, 300 MHz): δ 0.87 (t, *J* = 6.7, 3H, CH_3), 1.23-1.30 (m, 24H, $12CH_2$), 1.43 (s, 9H, $3CH_3$), 2.03 (app q, *J* = 6.9, 2H, $C\underline{H}_2CH_{alkene}$), 2.80 (br s, 1H, OH), 3.78 (d, *J* = 11.1, 3H, OCH_3), 3.79 (d, *J* = 11.1, 3H, OCH_3), 3.77-3.82 (m, 1H, $C\underline{H}NH$), 4.08-4.15 (m, 2H, CH_2O), 4.33 (ddd, *J* = 10.7, 7.8, 4.7, 1H, $C\underline{H}OH$), 5.01 (br d, *J* = 8.4, 1H, NH), 5.50 (dd, *J* = 15.4, 7.0, 1H, $C\underline{H}_{alkene}CH$), 5.75 (dt, *J* = 15.4, 6.6, 1H, $CH_2C\underline{H}_{alkene}$). ^{13}C-NMR ($CDCl_3$, 75 MHz): δ 14.2 ($CH_3$), 22.8 ($CH_2$), 28.5 ($3CH_3$), 29.2, 29.4, 29.5, 29.6, 29.7, 29.8 ($6CH_2$), 29.83 ($4CH_2$), 32.1, 32.5 ($2CH_2$), 54.7 (d, *J* = 5.9, $2OCH_3$), 55.1 (br s, CHNH), 66.8 (d, *J* = 5.7, CH_2O), 72.6 (CHOH), 78.8 (C), 128.6, 135.1 ($2CH_{alkene}$), 155.9 (CO).

***tert*-Butyl ((1*S*,2*R*,3*E*)-1-{[(dimethoxyphosphoryl)oxy]methyl}-2-hydroxynonadec-3-en-1-yl)carbamate (9)**

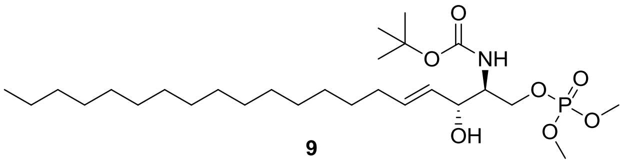

9

Obtained from 1-heptadecene (190 mg, 0.80 mmol) and **5** (65 mg, 0.20 mmol) in 25% yield. R_f: 0.28 (DCM/ethyl acetate, 1:1). ^{1}H-NMR ($CDCl_3$, 300 MHz): δ 0.87 (t, *J* = 6.8, 3H, CH_3), 1.25-1.38 (m, 26H, $13CH_2$), 1.43 (s, 9H, $3CH_3$), 2.03 (app q, *J* = 6.9, 2H, $C\underline{H}_2CH_{alkene}$), 2.76 (br s, 1H, OH), 3.78 (d, *J* = 11.1, 3H, OCH_3), 3.80 (d, *J* = 11.1, 3H, OCH_3), 3.74-3.82 (m, 1H, $C\underline{H}NH$), 4.08-4.16 (m, 2H, CH_2O), 4.33 (ddd, *J* = 10.7, 7.8, 4.7, 1H, $C\underline{H}OH$), 5.01 (br d, *J* = 7.8, 1H, NH), 5.50 (dd, *J* = 15.4,

7.0, 1H, $C\underline{H}_{alkene}CH$), 5.75 (dt, *J* = 15.4, 6.6, 1H, $CH_2C\underline{H}_{alkene}$). ^{13}C-NMR ($CDCl_3$, 75 MHz): δ 14.1 ($CH_3$), 22.7 ($CH_2$), 28.4 ($3CH_3$), 29.1, 29.3, 29.4, 29.5 ($4CH_2$), 29.6 ($2CH_2$), 29.7 ($5CH_2$), 31.9, 32.3 ($2CH_2$), 54.5 (d, *J* = 6.0, $2OCH_3$), 55.0 (d, *J* = 2.0, CHNH), 66.7 (d, *J* = 5.9, CH_2O), 72.5 (CHOH), 81.3 (C), 128.5, 135.0 ($2CH_{alkene}$), 155.7 (CO).

General procedure for the synthesis of final compounds 1-4

To a solution of the corresponding phosphate **6**-**9** (1 equiv) in DCM (5 mL/mmol), bromotrimethylsilane (8.3 equiv) was added at room temperature and the reaction mixture was stirred for 4 h. Then, the solvent was evaporated under reduced pressure. The resulting crude silyl ester was dissolved in methanol (5 mL/mmol) and the solution was stirred for an additional 10 minutes. Afterward, the solvent was removed under reduced pressure to afford the desired final products **1**-**4** without further purification.

(2*S*,3*R*,4*E*)-2-Amino-3-hydroxyhexadec-4-en-1-yl dihydrogen phosphate (1)

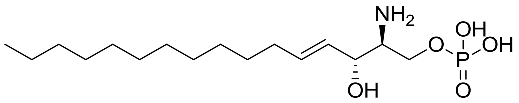

1

Obtained from **6** (15 mg, 31 µmol) in quantitative yield. R_f: 0.05 (DCM/ethyl acetate, 1:1). IR (ATR): ν 3340, 1615, 1044. ^{1}H-NMR (methanol-d_4, 500 MHz): δ 0.92 (t, *J* = 7.0, 3H, CH_3), 1.31-1.37 (m, 16H, $8CH_2$), 1.42-1.48 (m, 2H, $C\underline{H}_2CH_2CH_{alkene}$), 2.13 (app q, *J* = 7.0, 2H, $C\underline{H}_2CH_{alkene}$), 3.45 (qt, *J* = 4.1, 1H, $C\underline{H}NH_2$), 4.09-4.14 (m, 1H, ½CH_2O), 4.21-4.25 (m, 1H, ½CH_2O), 4.35 (app t, *J* = 5.6, 1H, $C\underline{H}OH$), 5.51 (dd, *J* = 15.5, 6.9, 1H, $C\underline{H}_{alkene}CH$), 5.92 (dt, *J* = 15.3, 6.7, 1H, $CH_2C\underline{H}_{alkene}$). ^{13}C-NMR (methanol-$d_4$, 125 MHz): δ 14.4 ($CH_3$), 23.7, 30.1, 30.4, 30.5, 30.6, 30.7, 30.75, 30.8, 33.1, 33.4 ($10CH_2$), 57.0 (d, *J* = 7.3, $CHNH_2$), 64.0 (d, *J* = 4.6, CH_2O), 70.6 (CHOH), 128.1, 137.0 ($2CH_{alkene}$). ^{31}P-NMR (methanol-d_4, 202 MHz): δ 2.99. $[\alpha]_D^{20}$ = −1.01 (c = 1.29, methanol). HRMS (ESI, *m/z*): calculated for $C_{16}H_{33}NO_5P$ ($[M-H]^-$): 350.2102, found 350.2100.

(2*S*,3*R*,4*E*)-2-Amino-3-hydroxyheptadec-4-en-1-yl dihydrogen phosphate (2)

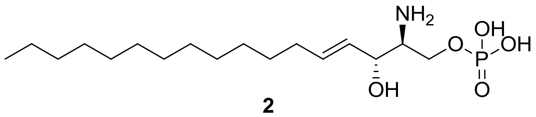

2

Obtained from **7** (16 mg, 32 µmol) in quantitative yield. R_f: 0.05 (DCM/ethyl acetate, 1:1). IR (ATR): ν 3432, 1625, 1175, 1049. ^{1}H-NMR (methanol-d_4, 500 MHz): δ 0.90 (t, *J* = 6.5, 3H, CH_3), 1.29-1.46 (m, 20H, $10CH_2$), 2.11 (app q, *J* = 7.0, 2H, $C\underline{H}_2CH_{alkene}$), 3.42 (qt, *J* = 4.1, 1H, $C\underline{H}NH_2$), 4.04-4.13 (m, 1H, ½CH_2O), 4.17-4.22 (m, 1H, ½CH_2O), 4.33 (app t, *J* = 5.5, 1H, $C\underline{H}OH$), 5.49 (dd, *J* = 15.4, 6.7, 1H, $C\underline{H}_{alkene}CH$), 5.89 (dt, *J* = 15.4, 6.6, 1H, $CH_2C\underline{H}_{alkene}$). ^{13}C-NMR (methanol-$d_4$, 125 MHz): δ 14.4 ($CH_3$), 23.7, 30.1, 30.4, 30.5, 30.6, 30.7, 30.72, 30.74, 30.8, 33.1, 33.4 ($11CH_2$), 57.1 (d, *J* = 7.1, $CHNH_2$), 64.1 (d, *J* = 4.2, CH_2O), 70.7 (CHOH), 128.1, 137.1 ($2CH_{alkene}$). ^{31}P-NMR (methanol-d_4, 202 MHz): δ 2.99. $[\alpha]_D^{20}$ = −1.53 (c = 1.30, methanol). HRMS (ESI, *m/z*): calculated for $C_{17}H_{35}NO_5P$ ($[M-H]^-$): 364.2258, found 364.2276.

(2*S*,3*R*,4*E*)-2-Amino-3-hydroxynonadec-4-en-1-yl dihydrogen phosphate (3)

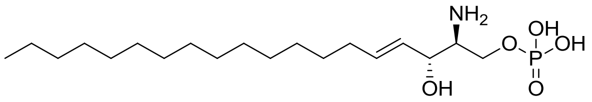

3

Obtained from **8** (16 mg, 31 µmol) in quantitative yield. R_f: 0.05 (DCM/ethyl acetate, 1:1). IR (ATR): ν 3345, 1612, 1465, 1167, 1051. ^{1}H-NMR (methanol-d_4, 500 MHz): δ 0.90 (t, J = 6.6, 3H, CH_3), 1.29-1.45 (m, 24H, 12CH_2), 2.11 (app q, J = 6.9, 2H, $C\underline{H}_2CH_{alkene}$), 3.43 (qt, J = 4.1, 1H, $C\underline{H}NH_2$), 4.06-4.15 (m, 1H, ½CH_2O), 4.19-4.26 (m, 1H, ½CH_2O), 4.33 (app t, J = 5.7, 1H, C$\underline{H}$OH), 5.49 (dd, J = 15.4, 6.7, 1H, $C\underline{H}_{alkene}CH$), 5.90 (dt, J = 15.0, 6.8, 1H, $CH_2C\underline{H}_{alkene}$). ^{13}C-NMR (methanol-$d_4$, 125 MHz): δ 14.4 ($CH_3$), 23.7, 30.1, 30.4, 30.5, 30.6 (5CH_2), 30.7 (2CH_2), 30.8 (4CH_2), 33.1, 33.4 (2CH_2), 57.0 (d, J = 7.4, $CHNH_2$), 64.2 (d, J = 4.5, CH_2O), 70.6 (CHOH), 128.0, 137.1 (2CH_{alkene}). ^{31}P-NMR (methanol-d_4, 202 MHz): δ 2.71. $[\alpha]_D^{20}$ = −1.64 (c = 1.22, methanol). HRMS (ESI, m/z): calculated for $C_{19}H_{39}NO_5P$ ($[M-H]^-$): 392.2571, found 392.2575.

(2*S*,3*R*,4*E*)-2-Amino-3-hydroxyicos-4-en-1-yl dihydrogen phosphate (4)

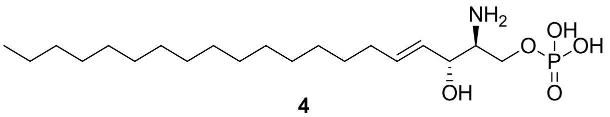

4

Obtained from **9** (12 mg, 22 µmol) in quantitative yield. R_f: 0.05 (DCM/ethyl acetate, 1:1). IR (ATR): ν 3366, 1607, 1466, 1183, 1042. ^{1}H-NMR (methanol-d_4, 300 MHz): δ 0.90 (t, J = 6.8, 3H, CH_3), 1.29-1.46 (m, 26H, 13CH_2), 2.11 (app q, J = 6.9, 2H, $C\underline{H}_2CH_{alkene}$), 3.40-3.46 (m, 1H, $C\underline{H}NH_2$), 4.05-4.11 (m, 1H, ½CH_2O), 4.21-4.24 (m, 1H, ½CH_2O), 4.33 (app t, J = 5.4, 1H, C$\underline{H}$OH), 5.49 (dd, J = 15.3, 6.7, 1H, $C\underline{H}_{alkene}CH$), 5.90 (dt, J =15.3, 6.6, 1H, $CH_2C\underline{H}_{alkene}$). ^{13}C-NMR (methanol-$d_4$, 75 MHz): δ 14.4 ($CH_3$), 23.7, 30.1, 30.4, 30.45, 30.6 (5CH_2), 30.7 (2CH_2), 30.8 (5CH_2), 33.1, 33.4 (2CH_2), 57.0 (d, J = 5.0, $CHNH_2$), 64.2 (d, J = 4.1, CH_2O), 70.6 (CHOH), 128.0, 137.1 (2CH_{alkene}). ^{31}P-NMR (methanol-d_4, 202 MHz): δ 2.78. $[\alpha]_D^{20}$ = −2.36 (c = 0.78, methanol). HRMS (ESI, m/z): calculated for $C_{20}H_{41}NO_5P$ ($[M-H]^-$): 406.2728, found 406.2738.

1.2. Purity Analyses for Final Compounds 1-4

Compound	Molecular Formula	MS (ESI) (m/z)	Retention time (min)
**1**	$C_{16}H_{34}NO_5P$	350.2 $[M-H]^-$	26.50
**2**	$C_{17}H_{36}NO_5P$	364.2 $[M-H]^-$	27.32
**3**	$C_{19}H_{40}NO_5P$	392.2 $[M-H]^-$	32.11
**4**	$C_{20}H_{42}NO_5P$	406.2 $[M-H]^-$	35.14

Supplementary References

1. Mezei, M. Simulaid: A simulation facilitator and analysis program. *J. Comput. Chem.* **31,** 2658–2668 (2010).

2. Manglik, A. *et al.* Structural Insights into the Dynamic Process of β2-Adrenergic Receptor Signaling. *Cell* **161,** 1101–11 (2015).

3. Yamamoto, T., Hasegawa, H., Hakogi, T. & Katsumura, S. Versatile synthetic method for sphingolipids and functionalized sphingosine derivatives via olefin cross metathesis. *Org. Lett.* **8,** 5569–5572 (2006).
